# Generation of orthotopic intracranial glioblastoma patient-derived xenograft models: insights into extrachromosomal DNA-driven MYC(N) and PDGFRA oncogene amplification and preliminary therapeutic evaluation

**DOI:** 10.1016/j.neo.2025.101233

**Published:** 2025-09-25

**Authors:** Thi-Anh-Thuy Tran, Sinae An, Junghyun Lim, Young-Hee Kim, Ahyeon Shim, Taewoo Han, Hawsan Kim, Sue-Jee Park, Yeong Jin Kim, Kyung-Sub Moon, In-Young Kim, Shin Jung, Chul Won Lee, Kyung-Hwa Lee, Ae Kyung Park, Tae-Young Jung

**Affiliations:** a Brain Tumor Research Laboratory, Chonnam National University Medical School and Hwasun Hospital, Hwasun, Republic of Korea; b Department of Neurosurgery, Chonnam National University Medical School and Hwasun Hospital, Hwasun, Republic of Korea; c Department of Pathology, Chonnam National University Medical School and Hwasun Hospital, Hwasun, Republic of Korea; d Department of Pharmacy, School of Pharmacy and Institute of New Drug Development, Jeonbuk National University, Jeonju, Republic of Korea; e INDNA, Hwaseong-si, Gyeonggi-do, Republic of Korea; f Department of Chemistry, Chonnam National University, Gwangju, Republic of Korea; g BioMedical Sciences Graduate Program (BMSGP), Chonnam National University Medical School, Hwasun, Republic of Korea

**Keywords:** Glioblastoma, PDX model, Extrachromosomal DNA, MYC(N) amplification, PDGFRA amplification, NK cell therapy

## Abstract

**Background:**

This study aimed to establish orthotopic intracranial patient-derived xenograft (PDX) models to investigate molecular and pathological features and to evaluate potential preclinical therapeutic approaches in glioblastoma (GBM).

**Methods:**

Fresh or cryopreserved patient tumor tissues were first expanded as subcutaneous PDXs and subsequently used to generate orthotopic intracranial PDXs. Tumor growth and similarity to patient tumors were assessed by magnetic resonance imaging (MRI), pathological analyses, and multi-omics profiling. A selected intracranial PDX was further used to evaluate the potential preclinical efficacy of natural killer (NK) cells combined with Avastin® and irinotecan. The cytotoxic effects of this combination were also examined in primary GBM cells obtained from the original tumor of the same PDX.

**Results:**

Subcutaneous PDX engraftment was successful in 13 out of 16 cases (81.3 %), and orthotopic intracranial PDXs were established from six of these with a 100 % success rate. Subcutaneous tumors expanded within 9 to 31 weeks, while intracranial tumors formed within 4 to 14 weeks. Subcutaneous growth was influenced by the Ki-67 index and the cryopreservation duration. Multi-omics analysis revealed extrachromosomal DNA (ecDNA)-driven amplifications of MYC(N), PDGFRA, CDK4, and MDM2 in PDXs from two patients. PDGFRA, CDK4, and MDM2 amplifications were consistent with those in the primary tumors, whereas MYC(N) amplification, initially minimal or absent in patient samples, was markedly enriched in the PDXs. Of the multiple PDXs from a single patient, the one PDX harboring ecDNA-driven MYCN amplification showed a greatly accelerated growth rate. Notably, PDXs containing ecDNA-driven MYC amplification exhibited a histological transformation toward primitive embryonal features. Combining NK cells with Avastin® and irinotecan enhanced cytotoxicity in vitro and prolonged survival in intracranial PDXs harboring ecDNA-driven MYC and PDGFRA amplifications.

**Conclusion:**

Intracranial PDX models were successfully established from cryopreserved GBM tissues through subcutaneous expansion. These models offer a clinically relevant platform for investigating GBM biology and evaluating the therapeutic efficacy of chemoimmunotherapy.

## Background

Mouse models are widely used for studying cancer pathology and developing effective therapies for various aggressive cancers, including glioblastoma (GBM). Standard cell-line xenograft models are commonly utilized; however, patient-derived xenograft (PDX) models—established by directly engrafting GBM tumor tissues or freshly isolated/short-time cultured primary GBM cells into immunodeficient or humanized mice—are frequently preferred in preclinical cancer research because they better capture the complex and heterogeneous biology of cancer [[1], [2], [3], [4], [5], [6], [7]]. Although subcutaneous PDX tumors reflect many GBM features, including heterogeneity and morphological/genetic variation, they fail to recapitulate the tumor microenvironments (TME) of the central nervous system (CNS), such as invasive growth and the presence of the blood-brain barrier (BBB). Consequently, orthotopic intracranial models are considered more clinically relevant for translational research, as they reproduce GBM's invasive nature and provide a superior platform for prognosis prediction, pathway discovery, and therapy evaluation [[8], [9], [10], [11]].

Direct generation of intracranial PDXs from fresh or short-term cultured primary patient cells is limited by restricted tissue availability, variable cell viability, serum-free culture requirements, and inconsistent or delayed tumor formation. To overcome these challenges, an alternative strategy has been developed in which cryopreserved GBM tissues are first expanded as subcutaneous PDXs before intracranial implantation. This approach increases tissue availability and tumor engraftment rates while preserving the cellular and genetic characteristics of the original tumors [[12], [13], [14], [15]].

Extrachromosomal DNA (ecDNA), a large, circular, and self-replicating DNA carrying various gene fragments, has gained increasing attention as a major driver of oncogene amplification in many cancers, including GBM. Studies have shown that ecDNAs contribute to rapid tumor evolution, intratumoral genetic heterogeneity, resistance to cancer therapies, poor clinical outcomes, and remodeling of TME [[16], [17], [18], [19], [20], [21], [22], [23], [24]]. A longitudinal study demonstrated that oncogenic ecDNA elements in primary tumors are amplified in both recurrent tumors and their derivative neurosphere and PDX models, which indicates that these models recapitulate tumor recurrence [25].

In this study, we established orthotopic intracranial GBM PDX models from fresh or cryopreserved patient tissues via subcutaneous expansion. Multi-omics profiling revealed ecDNA-driven amplifications of oncogenes, including MYC(N), PDGFRA, and CDK4/MDM2. We then evaluated the efficacy of natural killer (NK) cells in combination with Avastin® and irinotecan against primary GBM cells in vitro and in intracranial PDX models harboring ecDNA-driven MYC and PDGFRA amplifications. These models faithfully preserve key molecular alterations and provide a preclinical platform for evaluating chemoimmunotherapy in GBM.

## Methods

### Patient samples, cryopreservation, and animals

Surgical specimens from sixteen GBM patients were obtained directly from the operating theater at the Neurosurgery Department of Chonnam National University Hwasun Hospital (CNUHH). Each tumor specimen was divided into two portions under sterile conditions. One portion was fixed in formalin and embedded in paraffin for pathological and genetic analysis. The other portion was immediately transported to the laboratory, where it was cut into small fragments (∼2 × 2 × 2 mm³) for immediate implantation or cryopreservation. For cryopreservation, tumor fragments were placed in sterile cryovials containing freezing medium (10 % dimethyl sulfoxide [DMSO] in fetal bovine serum [FBS]), stored at −80°C overnight in a controlled-rate freezing container, and subsequently transferred to liquid nitrogen for long-term storage.

Female NOD/SCID IL-12Rγnull (NSG) mice and BALB/c nude mice (6–8 weeks old) were obtained from Jackson Laboratory (Bar Harbor, MA, USA) and Orient Bio (Iksan, Korea), respectively. Mice were bred and maintained under specific pathogen-free conditions. Anesthesia was induced by intraperitoneal (IP) injection of a 2:1 mixture of Zoletil® (Virbac Laboratories, Carros, France) and Rompun® (Bayer Korea, Anshan, Korea) at a dose of 1.5 mL/kg. All animal care, experimental procedures, and euthanasia were conducted in accordance with the guidelines of the Chonnam National University Institutional Animal Care and Use Committee (CNU-IACUC—H-2017-51).

### Histological and immunohistochemical (IHC) analysis

All specimens were fixed in 10 % neutral-buffered formalin (Corebiotech, Korea) and embedded in paraffin to generate formalin-fixed paraffin-embedded (FFPE) blocks. Sections (3 µm) were prepared for hematoxylin and eosin (H&E) and immunohistochemical staining.

For H&E, sections were deparaffinized with xylene and rehydrated through a graded alcohol series. Tissue sections were stained with hematoxylin for 3 minutes, rinsed in water, and treated with a differentiator for 1 minute. Following another water wash, sections were treated with a bluing reagent for 1 minute. Eosin counterstaining was performed for 45 seconds. The slides were then dehydrated in 95 % and 100 % ethanol, cleared in xylene, and mounted with coverslips using a standard mounting medium.

For IHC, sections were deparaffinized with xylene and rehydrated through a graded alcohol series. Endogenous peroxidase activity was blocked, and antigen retrieval was conducted using BOND epitope retrieval solutions: citrate buffer (ER1, pH 6) for 20 minutes, Tris-EDTA buffer (ER2, pH 9) for 20 minutes, or Tris-EDTA buffer (CC1, pH 9) for 48 minutes. Non-specific binding was blocked using a protein-blocking buffer. Sections were incubated with primary antibodies against anti-human GFAP (1:400; clone 6F2; DAKO, Denmark), anti-human P53 (1:100; clone DO-7; DAKO, Denmark), anti-human Ki-67 (RTU; clone 30-9; Roche, Switzerland), anti-human Chromogranin A (CGA; 1:300 dilution; clone DAK-43; DAKO, Denmark), anti-human Synaptophysin (1:100 dilution; clone 27G12; LEICA, Germany), anti-human Nestin (1:400 dilution; clone 10C2; CHEMICON, Korea), CDK4 (1:200 dilution, clone ab7955, Abcam, United Kingdom), and anti-human CD45RO (LCA; 1:100 dilution; clone 2B11+PD7/26; DAKO, Denmark). Detection was performed using the BOND Polymer Refine Detection Kit (Leica Biosystems, Germany) with diaminobenzidine (DAB) as the chromogen. Immunohistochemical staining was performed using an automated immunostainer (Bond-III, Leica Biosystems, Nussloch, Germany).

Slides were scanned using the Zeiss AxioScan.Z1 or Aperio ScanScope System, and images were processed using Zen Blue software (Zeiss, Germany). The Ki-67 proliferation index was assessed using Roche uPath Image Analysis Algorithms (Roche Diagnostics, Rotkreuz, Switzerland). All aspects of the histopathological examination, including review of tissue slides, interpretation of immunohistochemical staining, and selection of appropriate hotspots for image analysis algorithms, were conducted by an experienced pathologist (KHL).

### Targeted sequencing and MGMT promoter methylation analysis in sixteen patient tumor samples

Tumor regions with >80 % purity were carefully dissected from FFPE blocks. The dissected tissue sections (10 µm) underwent deparaffinization and rehydration. Genomic DNA extraction was performed using the QIAamp DNA FFPE Tissue Kit (Qiagen, Hilden, Germany). DNA fragmentation was performed using the SureSelect Enzymatic Fragmentation Kit (Agilent Technologies) for subsequent bisulfite conversion and/or library preparation.

Targeted next-generation sequencing (NGS) was performed using a customized cancer panel (ONCOAccuPanel DNA, NGeneBio, Seoul, Korea) [26], designed to detect single nucleotide variants (SNVs) and structural variations across 323 cancer-associated genes. DNA libraries were prepared following the manufacturer’s protocol. DNA hybridization capture was conducted using biotin-labeled RNA probes specific to target regions. Hybridized DNA fragments were captured using Dynabeads MyOne Streptavidin T1 (Thermo Fisher Scientific, Waltham, MA, USA). Size selection and purification were performed using Agencourt AMPure XP beads (Beckman Coulter, Brea, CA, USA). Sequencing was performed on the Illumina NextSeq 500/550 platform (Illumina, San Diego, CA, USA) using the NextSeq 500/550 Mid Output Kit v2.5 with a paired-end read configuration. Raw reads were aligned to the human reference genome (hg19) using BWA-MEM (v0.7.17). Variant calling was performed using GATK (v4.0.6.0), SAMtools (v1.3.1), and VarScan (v2.4.3). Copy number variations (CNVs) were analyzed using CNVkit (v0.9.6), while gene fusion events were detected using Breakmer. Microsatellite instability (MSI) analysis was conducted using MSIseq, MSIpred, and mSINGs. Tumor mutational burden (TMB) was also assessed. Variant annotation and interpretation were performed using multiple databases, including ClinVar, dbNSFP, dbSNP, 1000Genomes, ESP6500, ExAC, gnomAD, KOEX, and KRGDB. Variants with a variant allele frequency (VAF) ≥ 3 % were reported. CNV amplifications and deletions were defined as ≥ 5 copies and ≤ 0 copies, respectively. The sensitivities for detecting SNVs/indels, translocations, and CNVs were 99.6 %, 100 %, and 96.6 %, respectively, with 100 % specificity. Variant classification was performed using the Cancer Genome Interpreter (CGI) and CIViC databases.

For MGMT promoter methylation analysis, bisulfite modification of genomic DNA was conducted using the EZ DNA Methylation-Lightning Kit (Zymo Research, Irvine, CA, USA). A total of 20 µL of extracted DNA was treated with 130 µL of Lightning Conversion Reagent, and bisulfite conversion was performed under the following thermal cycling conditions: 98°C for 8 min, followed by 54°C for 60 min, with optional storage at 4°C for up to 20 h. The bisulfite-converted DNA was purified using Zymo-Spin IC Columns and eluted in 10 µL of M-Elution Buffer. Methylation-specific PCR (MSP) was performed to assess MGMT promoter methylation status. The following primer sets were used: Unmethylated (forward: 5′-TTTGTGTTTTGATGTTTGTAGGTTTTTGT-3′, reverse: 5′-AACTCCACACTCTTCCAAAAACAAAACA-3′); Methylated (forward: 5′-TTTCGACGTTCGTAGGTTTTCGC-3′, reverse: 5′-GCACTCTTCCGAAAACGAAACG-3′). PCR conditions included an initial denaturation at 95°C for 5 min, followed by 40 cycles of 94°C for 45 s, 59°C for 45 s, and 72°C for 60 s, with a final extension at 72°C for 5 min. Control DNA from normal lymphocytes was treated with SssI methyltransferase (New England Biolabs, Beverly, MA, USA) for the methylated control, while untreated lymphocyte DNA served as the unmethylated control. PCR products were separated on a 4 % agarose gel, stained with Midori Green Advance (NIPPON Genetics, Japan), and visualized under UV light. The expected band sizes were 81 bp (methylated) and 93 bp (unmethylated).

### Generation of a heterotopic subcutaneous PDX mouse model for tumor expansion

The original GBM patient cells were expanded in a subcutaneous mouse model using fresh or cryopreserved GBM tissue, following previously described methods [12,13,27]. NSG mice were anesthetized for subcutaneous implantation, and fresh or cryopreserved GBM tissues were chopped into small fragments (approximately 2 × 2 × 2 mm³). The tumor fragments were washed with phosphate-buffered saline (PBS; Goldbio, USA), and necrotic tissues were removed. A subcutaneous pocket was created using sterile scissors, and small tumor fragments (approximately 0.3 g) were implanted into the pocket with or without 200 µL of Matrigel™ Matrix (Corning #354248) using sterile forceps. The incision was sutured and disinfected with a 10 % povidone-iodide solution (Betadine, Sungkwang Pharmaceutical Co Ltd, Bucheon, South Korea).

Tumor growth was monitored weekly, and tumor volume was calculated using the ellipsoid volume formula: *V* = 4/3π [(length x width x height)/8]. Mice were euthanized when individual tumor volumes reached approximately 1,500 mm³. The harvested tumors were subsequently processed for genetic analysis, cryopreservation for tissue banking, further subcutaneous expansion, or generation of orthotopic intracranial PDX models.

### Generation of an orthotopic intracranial PDX mouse model for therapeutic confirmation

To generate an intracranial GBM PDX model (P2), a single-cell suspension derived from established subcutaneous PDX tumors (P1) was injected into the brains of NSG or BALB/c nude mice. Each sample was typically implanted into 2–3 mice.

Subcutaneous PDX tumors were collected and washed with RPMI-1640 medium (Gibco, NY, USA), supplemented with 10 % FBS and 1 % penicillin/streptomycin (P/S). Tumors were then minced into small fragments (approximately 3–4 mm) using a sterile scalpel and dissociated into single-cell suspensions using a tumor dissociation kit (Miltenyi Biotech, Germany). The resulting suspension was filtered through 70 µm and 40 µm cell strainers (Falcon, USA) to remove large debris, followed by treatment with a debris removal solution (Miltenyi Biotech, Germany). Erythrocytes were eliminated using a red blood cell lysis solution (Miltenyi Biotech, Germany).

For intracranial injection, 5 × 10⁵ single tumor cells were resuspended in 5 µL of PBS and injected stereotactically into the right striatum of each mouse at a flow rate of 1 µL per minute using a Harvard Apparatus Pump 11 Elite infusion system (Harvard Apparatus, Holliston, MA, USA) connected to a Hamilton syringe. The stereotactic coordinates used were 1 mm posterior and 2 mm lateral from the bregma, with an injection depth of 4 mm from the cortical surface. Tumor formation and progression were monitored using magnetic resonance imaging (MRI) with T2-weighted and contrast-enhanced T1-weighted sequences. Image analysis was performed using RadiAnt DICOM Viewer 2024.1 software.

### Comprehensive genomic, transcriptomic, and epigenomic analysis of two patient primary tumors and their corresponding PDX models

Genomic DNA was extracted from blood and tissue samples, and libraries for whole genome sequencing (WGS), whole exome sequencing (WES), and methylome sequencing were subsequently constructed using the single-strand–based SRSLY NanoPlus DNA NGS Library Preparation Base Kit (Claret Bioscience, USA), according to previously described methods [28]. For RNA-sequencing (RNA-seq), total RNA was extracted from tissue samples, and 100 ng of total RNA was subjected to a sequencing library construction using the Agilent SureSelect RNA Direct kit and the Agilent SureSelect XT Human All Exon V6+UTRs Kit Final libraries were sequenced on the Illumina NovaSeq 6000 platform (Macrogen Inc., Korea) using paired-end 150 bp reads for WGS, WES, and methylome sequencing, and paired-end 100 bp reads for RNA-seq.

For the preprocessing of WGS and WES data, TrimGalore (v0.6.10) was employed to remove adapter sequences and discard reads shorter than 50 bp. To filter out mouse sequences, BBSplit was used to align the sequence reads to both the human (GRCh38) and mouse genomes (GRCm39), separating them accordingly. Ambiguous sequence reads (those aligning to both genomes) were discarded. Human-specific reads were aligned to the human genome (GRCh38) using BWA-MEM (v0.7.17). Picard tools were utilized for deduplication and indexing. Base Quality Score Recalibration (BQSR) was performed using GATK (v4.2.6.1). For the detection of mutations, VCF files were generated from WES data using the GATK mutect2 in "normal-tumor" paired mode with an option of germline resource set to af-only-gnomad.hg38.vcf.gz from the GATK resource bundle, and raw mutation calls were filtered with the GATK FilterMutectCalls. Copy number variations (CNVs) were identified from recalibrated BAM files from WES data using CNVkit (v0.9.11), with a pooled reference generated by combining two normal samples. To detect ecDNAs, we applied the Gene-level Circular Amplicon Prediction (GCAP) tool (v1.2.0) [29] to recalibrated BAM files from WES data and the AmpliconSuite-pipeline (v1.3.5) [30,31] to preprocessed fastq files from WGS data containing human-specific reads.

For RNA-seq data analysis, raw reads were trimmed using TrimGalore with default options and mouse reads were filtered out with BBSplit as described above. Then, human sequences were quantified for gene or transcript abundance using Salmon (v1.10.1) [32]. To detect differentially expressed genes (DEGs), only genes with a count of at least 10 in more than two samples were selected, and DEG analysis was performed using the DESeq2 R package between P1 vs. P0, P2 vs. P0, and P2 vs. P1 in a paired-sample design. Enrichment analysis on DEGs was performed with the DAVID Functional Annotation Tool (https://david.ncifcrf.gov/) using the annotation category of GOTERM_BP_DIRECT. To further assess the enrichment of molecular pathways in individual samples, we employed gene set variation analysis (GSVA) [33] with hallmark gene sets using log_2_-transformed variance-stabilizing transformation (VST)-normalized gene expression values.

For the analysis of methylome data, raw reads were trimmed with TrimGalore using the options "–clip_R1 8, –three_prime_clip_R1 8, –clip_R2 8, and –three_prime_clip_R2 8". Trimmed reads were aligned to the mouse genome (GRCm39) with Bismark (v0.24.0), and the reads aligned to the mouse genome were removed. Subsequent alignment to the human genome (GRCh38), deduplication, and strand-specific methylation calling were performed with Bismark. Subsequently, we discarded the CpG sites that had coverage below 10 reads or > 99.9th percentile of coverage in each sample. Finally, only the CpG sites commonly detected within the Twist methylome target regions across all samples were retained for further analyses. Differentially methylated CpG sites were identified using Fisher’s exact test implemented in the methylKit R package [34], by comparing P1 vs. P0 or P2 vs. P0 within each tumor origin. Significance was defined as a methylation difference greater than 50 % and a false discovery rate (FDR)-adjusted *p*-value < 0.05.

To investigate the negative correlation between gene expression and promoter methylation for each Ensembl Canonical transcript, Pearson's correlation coefficient was calculated using expression and methylation levels averaged within the given promoter region in P0, P1, and P2 samples from each patient.

### Natural killer (NK) cell expansion and treatment schedule on orthotopic GBM PDX model

Following previously described protocols, NK cell expansion was performed using K562-OX40L-mb-IL18/IL21 feeder cells [34,35]. Peripheral blood mononuclear cells (PBMCs), collected from healthy donors by using Lymphoprep solution (Axis-Shield, Dundee, UK), were co-cultured with gamma-irradiated (100 Gy) K562-OX40L-mb-IL-18/IL-21 feeder cells in RPMI-1640 medium (Welgene, Daegu, Korea) complemented by 10 % of FBS (Gibco, NY, USA), 1 % of penicillin-streptomycin (Gibco, NY, USA) and 1 % of l-glutamine (Welgene, Daegu, Korea). NK cells were cultured with recombinant human interleukin IL-2 and IL-15 (PeproTech, NJ, USA). Recombinant IL-2 (10 IU/mL) was initially added to the cell culture medium until day 7. From day 7, recombinant IL-2 (100 IU/mL) and IL-15 (5 ng/mL) were added to the cell culture medium. The cytokine-containing culture medium was refreshed every 2–3 days. By day 14, NK cells with a purity exceeding 90 % were collected for “in vivo” use. NK cell purity was confirmed by flow cytometry. In general, expanded NK cells were stained with APC-conjugated anti-human CD56 antibody (clone B159, BD Biosciences, USA) and FITC-conjugated anti-human CD3 antibody (clone UCHT1, BD Biosciences, USA) for 30 minutes at 4°C. NK cell purity was assessed by analyzing the percentage of the CD56^+^CD3^-^ population using a Calibur flow cytometer (BD Biosciences, USA). Data acquisition was performed with FlowJo v10 software (TreeStar, San Carlos, CA, USA).

The orthotopic GBM PDX model was employed to evaluate the therapeutic efficacy of NK cells in combination with GBM standard treatments such as Avastin® and irinotecan on NSG mice. MRI monitored tumors, and treatment commenced when tumors were detectable on T2-weighted images on day 37. Mice-bearing intracranial tumors were treated with Avastin® (5 mg/ kg, Roche, Basel, Switzerland) and irinotecan (60 mg/ m^2^, Inno.N, Seoul, Korea) every four days, with a total of three doses administered via intraperitoneal (IP) injection. Expanded NK cells (2 × 10^7^ cells/ injection) were administered one day after each Avastin® plus irinotecan treatment. Tumor size was assessed pre- and post-treatment by MRI using T2-weighted images and analyzed using the RadiAnt DICOM Viewer 2024.1 software.

### Functional assays with primary GBM cells

Cryopreserved GBM3 tumor fragments were thawed, washed with PBS, and digested with 0.25 % collagenase type IV (Gibco, USA) at 37°C for 1 h with gentle agitation every 15 min. The dissociated cells were washed with RPMI medium supplemented with 10 % FBS and 1 % penicillin/streptomycin, sequentially filtered through 70-µm and 40-µm strainers (Falcon, USA), and erythrocytes were removed using a red blood cell lysis buffer (Miltenyi Biotec, Germany).

Cytotoxicity of expanded NK cells against primary GBM3 cells was assessed using the CytoTox 96 Nonradioactive LDH Cytotoxicity Assay (Promega, USA). Primary GBM3 cells, pretreated or not with Avastin® (500 µg/mL) and irinotecan (50 µg/mL) for 24 h, were plated at 4 × 10⁴ cells/ well in 96-well plates and co-cultured with NK cells at effector-to-target (E:T) ratios of 1:1 or 2.5:1 for 5 h at 37°C under 5 % CO₂. Supernatants were collected, and LDH release was quantified to calculate percent cytotoxicity according to the formula:

$$\%\;\text{Cytotoxicity}=\frac{\left(\text{Experimental}\;-\;\text{Effector}\;\text{Spontaneous}\;-\;\text{Target}\;\text{Spontaneous}\right)}{\left(\text{Target}\;\text{Maximum}\;-\;\text{Target}\;\text{Spontaneous}\right)}\times \;100$$

Cell viability was determined using the CCK-8 assay (Dojindo, USA). Primary GBM3 cells (5 × 10³/well) were seeded in 96-well plates, pretreated or not with Avastin® (500 µg/mL) and irinotecan (50 µg/mL) for 24 h, and then co-cultured with NK cells at E:T ratios of 1:1 or 2.5:1 for an additional 24 h. CCK-8 reagent (10 µL/well) was added, incubated for 4 h, and absorbance was measured at 450 nm using a SpectraMax i3x microplate reader (Molecular Devices, USA). Cell viability was calculated as:

$$\%\;\text{Cell}\;\text{viability}=\frac{\left(\text{Optical}\;\text{density}\;\left(\text{OD}\right)\;\text{experiment}\;-\;\text{OD}\;\text{blank}\right)}{\left(\text{OD}\;\text{control}\;-\;\text{OD}\;\text{blank}\right)}\times \;100$$

where experimental, blank, and control groups represent wells containing NK plus primary GBM3 cells pretreated or not with Avastin® and irinotecan for 24 h, medium only, and untreated tumor cells only, respectively.

Cytokine production was assessed by enzyme-linked immunosorbent assay (ELISA). Primary GBM3 cells (4 × 10⁴/ well) were pretreated or not with Avastin® (500 µg/mL) and irinotecan (50 µg/mL) for 24 h, co-cultured with NK cells at an E:T ratio of 2.5:1 for 24 h, and supernatants were collected. IFN-γ and TNF-α levels were measured using OptEIA ELISA kits (BD Biosciences). NK cells alone served as negative controls, and medium alone as a blank.

Degranulation was evaluated using the CD107a assay. Primary GBM3 cells (4 × 10⁴/ well), pretreated or not with Avastin® (500 µg/mL) and irinotecan (50 µg/mL) for 24 h, were co-cultured with NK cells at E:T ratios of 0.5:1, 1:1, or 2.5:1 in the presence of PE-conjugated anti-CD107a antibody (clone H4A3, BD Biosciences, USA). Monensin and brefeldin A (BD Biosciences, USA) were added after 1 h of incubation, and cultures were continued for an additional 4 h. NK cells were subsequently stained with APC-conjugated anti-human CD45 (clone HI30, BD Biosciences) and FITC-conjugated anti-human CD3 (clone UCHT1, BD Biosciences). The percentage of CD107a⁺ NK cells was determined using a CytoFLEX S flow cytometer (Beckman, USA) and analyzed with FlowJo v10 software (TreeStar, USA). PMA/Ionomycin (cell activation cocktail without Brefeldin A, Biolegend, USA) was used as a positive control.

### Statistical analysis

Statistical analyses were performed using GraphPad Prism 10.2.3 (GraphPad Software, San Diego, CA, USA) or R (https://www.r-project.org/). Differences between two groups were assessed using the Wilcoxon rank-sum test or Fisher’s exact test, while multiple groups were compared using one-way ANOVA. Survival curves were analyzed using the Kaplan–Meier method with log-rank test. A *p*-value < 0.05 was considered statistically significant.

## Results

### Overview of the generation of PDX mouse models and the molecular characteristics of sixteen GBM patient tumor samples

**Fig. 1:**
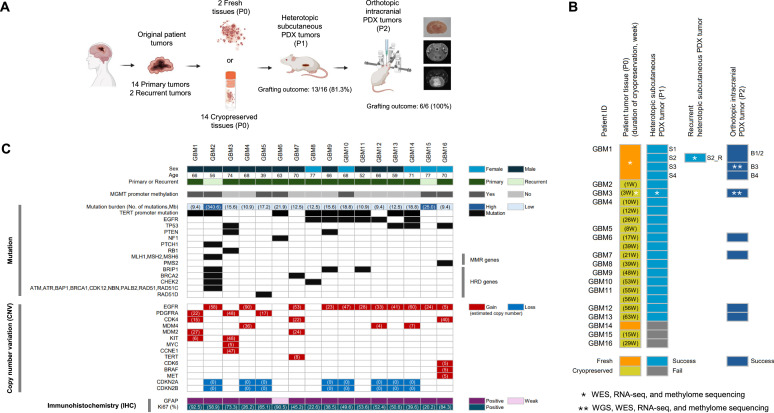
Overview of the generation of glioblastoma (GBM) patient-derived xenograft (PDX) model and characterization of patient tumors. (A) Schematic diagram illustrating the steps for generating PDX mouse models from GBM patient tumor tissues. (B) Summary of engraftment outcomes for subcutaneous and intracranial PDX models derived from sixteen patient tumors. (C) Clinical, histopathological, and molecular characteristics of patient tumors. Figure 1A was created using Biorender (http://biorender.com/).

A general protocol for generating PDX mouse models from GBM patient tumor tissues is presented in Fig. 1**A** and **Additional file 1: Figure S1**. All patient tumors were classified as GBM, IDH-wildtype, CNS WHO grade 4 according to the 2021 WHO classification [36]. To establish subcutaneous PDX models, we used fresh or cryopreserved GBM tumor specimens from sixteen GBM patients (from GBM1 to GBM16), including two recurrent and fourteen primary tumors. Two recurrent tumors (GBM2 and GBM15) were obtained from GBM patients who were treated with standard radiotherapy and temozolomide, with or without bevacizumab plus irinotecan. To establish orthotopic PDX models, subcutaneous PDX tumors were harvested, dissociated into single tumor cells, and implanted intracranially into NSG or BALB/c nude mice. Overall engraftment success rates were 81.3 % (13 out of 16 patients) and 100 % (6 out of 6 patients) in heterotopic subcutaneous and orthotopic intracranial PDX models, respectively. Detailed information on the generated PDX models is summarized in Fig. 1**B** and **Additional file 1: Table S1**. In GBM1, four subcutaneous PDX tumors (designated as **S1–S4**) were generated from a fresh patient tumor sample. In GBM4, GBM6, and GBM11 samples, multiple tumor samples with different cryopreservation durations were used to generate subcutaneous PDXs. Subsequent orthotopic intracranial PDXs were established in six patients, where subcutaneous PDXs yielded suitable tissue and sufficient mouse availability for orthotopic implantation. Comprehensive genomic, transcriptomic, and epigenomic analyses were performed on the primary and PDX tumors from two patients, GBM1 and GBM3, as indicated by asterisks in Fig. 1**B**.

Histopathological and molecular characterization of patient tumors was conducted, including targeted genomic analysis using ONCOAccuPanel [26] (Fig. 1**C**). MGMT promoter methylation was detected in seven samples. Consistent with previous studies [3,37], a higher mutation burden (> 23 mutations/Mb) was observed in recurrent tumors. The highest tumor burden was observed in GBM2, which harbored multiple mutations in mismatch repair (MMR) genes (MLH1, MSH2, MSH6) and homologous recombination deficiency (HRD)-related genes (BRCA1/2, ATM, ATR, BAP1, BRIP1, CDK12, CHEK2, NBN, PALB2, RAD51, RAD51C). TERT promoter mutations were the most prevalent, observed in nine samples (9/16, 56.3 %). In the CNV analysis, gain of EGFR was the most frequent event (11/16, 68.8 %), followed by loss of CDKN2A and CDKN2B (8/16, 50.0 %) and gain of PDGFRA, CDK4, or MDM4 (3/16, 18.8 %). IHC analysis revealed strong GFAP expression in most specimens, with Ki-67 proliferation indices ranging from 20.2 % to 92.5 %.

### Establishment of subcutaneous PDX model and influence of patient age and cryopreservation on engraftment

**Fig. 2:**
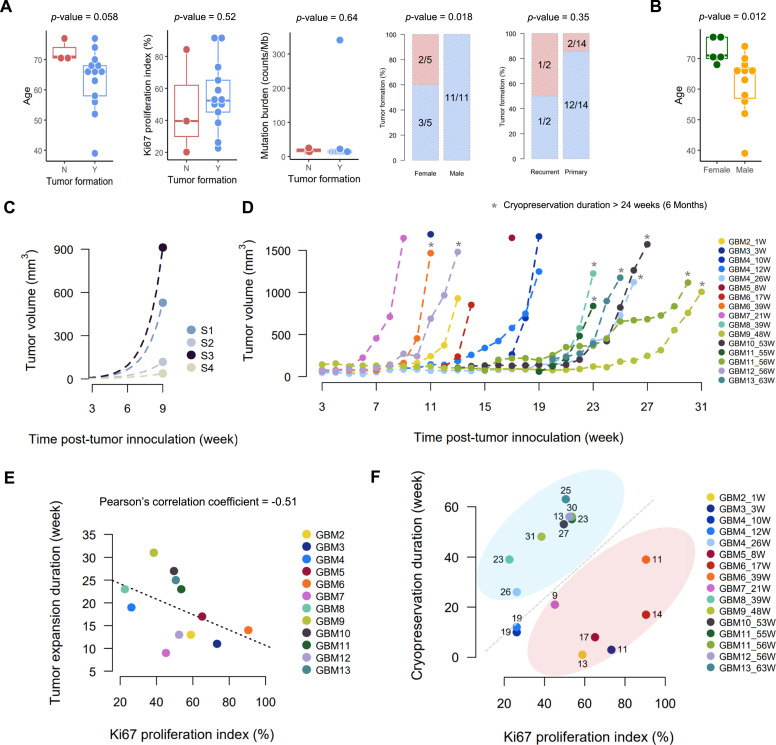
Tumor formation and growth in heterotopic subcutaneous PDX mouse models. (A) Analysis of factors influencing subcutaneous PDX engraftment success, including patient age, Ki-67 proliferation index, tumor burden, gender, and tumor type. (B) Distribution of patient age stratified by gender. (C) Tumor sizes monitored with a caliper at 9 weeks after inoculation (points) and hypothetical growth curves modeled with exponential functions (dashed lines) in four subcutaneous PDXs generated from the GBM1 patient tumor. (D) Tumor sizes weekly monitored with a caliper in 12 additional successful PDXs (GBM2 to GBM13). Tumor volume was calculated using the standard ellipsoid volume formula: *V* = 4/3π [(length x width x height)/8]. Mice were euthanized when tumor volume reached approximately 1,500 mm³. (E) Correlation between tumor expansion duration and Ki-67 proliferation index in 12 subcutaneous PDXs derived from cryopreserved GBM tissues. For GBM4, GBM5, and GBM11, expansion durations were averaged across multiple PDXs. (F) Relationship between cryopreservation duration, Ki-67 index, and tumor expansion time. Numbers next to each data point indicate the duration of tumor expansion (in weeks) for each PDX. GBM1–GBM13: patient identification numbers; W: week.

Of the sixteen patient tumors (P0), we successfully established heterotopic subcutaneous PDX models (P1) in thirteen patients, one from a fresh tumor (GBM1) and twelve from tumors that had been cryopreserved for 1 to 63 weeks (GBM2 – GBM13) (Fig. 1**B**). Successful engraftment of PDX was defined when the tumor reached a volume of 500 mm³ within eight months. When examining the impact of clinical and pathological factors on the success of subcutaneous PDX tumor formation, we observed a trend toward an association between older patient age and engraftment failure, with a marginal statistical significance (Wilcox rank sum test *p*-value = 0.058) (Fig. 2**A**). However, this association requires further validation in a larger cohort. A previous study reported no significant relationship between clinical characteristics and the success of viable flank xenograft formation in GBM [3]. In our analysis, patient sex appeared to influence engraftment success (Fisher’s exact test *p*-value = 0.018); however, the higher failure rate observed in female-derived samples may be confounded by the older age distribution within this group (Wilcox rank sum test *p*-value = 0.012) (Fig. 2**B**). Other factors, including Ki-67 proliferation index, mutation burden, and tumor recurrence, showed no significant association with engraftment success.

The time required for tumor expansion in successfully established subcutaneous PDX models ranged from 9 to 31 weeks (**Additional file 1: Table S1**). In GBM1, multiple subcutaneous PDXs (S1-S4) were generated, revealing considerable variations in their growth rates (Fig. 2**C**). To evaluate the impact of cryopreservation duration on tumor formation, we investigated tumor growth of subcutaneous PDXs generated from cryopreserved specimens and observed that PDXs derived from tumors cryopreserved for > 24 weeks exhibited relatively slower growth (Fig. 2**D**).

Additionally, the Ki-67 proliferation index of the original patient tumor was negatively correlated with the duration of tumor expansion (Fig. 2**E**), consistent with a recent study that reported a significant relationship between a high Ki-67 index and successful PDX engraftment in breast cancer [38]. Accordingly, we speculated that both the Ki-67 index and the cryopreservation duration may impact the PDX tumor engraftment period. Indeed, when the two variables were plotted together, the PDX samples were clearly stratified into two groups with prolonged or shortened expansion time (Fig. 2**F**).

### Engraftment of expanded heterotopic tumors into orthotopic PDX models

**Fig. 3:**
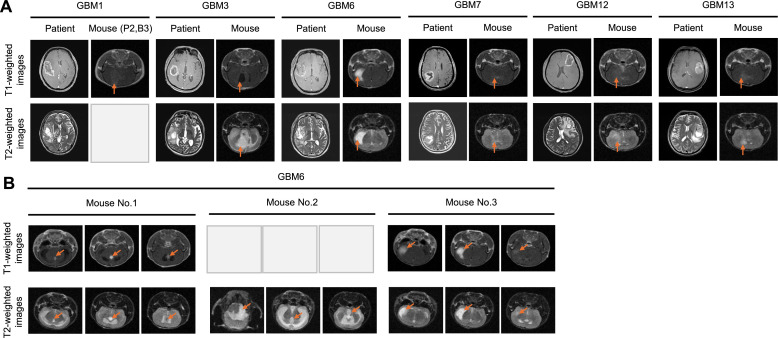
Comparison of tumor formation in GBM patients and corresponding intracranial PDX models by magnetic resonance imaging (MRI). (A) T1-weighted and T2-weighted MRI images confirming tumor formation in patients and corresponding intracranial PDX models from six patient samples (GBM1, GBM3, GBM6, GBM7, GBM12, and GBM13) out of 13 successful subcutaneous tumors. (B) MRI images showing variability within tumor growth locations in intracranial PDX models from GBM6. Orange arrows indicate PDX tumors formed in the mouse brain.

Orthotopic intracranial PDX models were successfully formed with all six selected subcutaneous PDX tumors (Fig. 1**B**). The tumor formation occurred between 4 and 14 weeks (**Additional file 1: Table S1**). MRI images of the original patient tumors showed indistinct tumor boundaries that were similarly mimicked in the intracranial PDXs (Fig. 3**A**). PDX tumor growth in GBM1, GBM3, GBM7, GBM12, and GBM13 showed diffuse infiltration localized near the tumor injection site. However, PDX tumors of GBM6 demonstrated migration of GBM cells into distant brain regions extending from the injection site (Fig. 3**B**). In Mouse No. 2 of GBM6, tumor cells spread via the cerebrospinal fluid (CSF) into the ventricles and cerebellar subarachnoid space. In Mouse No. 1, tumor infiltration occurred along white matter tracts into the corpus callosum, while in Mouse No. 3, the spread was along white matter tracts to the insular area..

### Increased cellular compactness in PDX tumors and acquisition of primitive embryonal features in GBM3

**Fig. 4:**
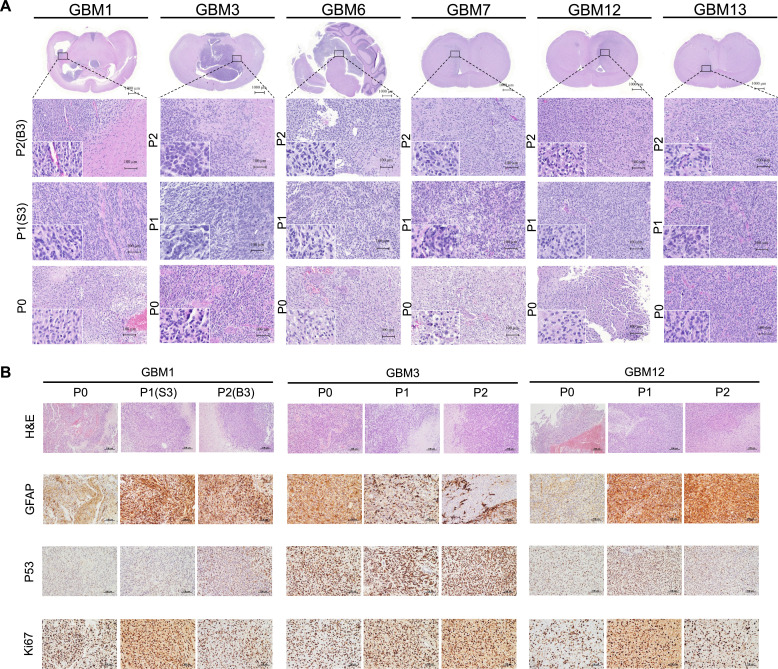
Histopathological comparison of patient tumors and corresponding subcutaneous and intracranial PDX models. (A) Hematoxylin and Eosin (H&E) staining comparing tumor morphology of patient tumors and their corresponding PDX tumors from six patients (GBM1, GBM3, GBM6, GBM7, GBM12, and GBM13). (B) Immunohistochemistry (IHC) staining analyzing the expression of GBM diagnostic marker (GFAP) and prognostic markers (p53 and Ki-67) in tumors from three patients (GBM1, GBM3, and GBM12).

Histological and IHC analyses were performed to compare the pathological characteristics of primary tumors with those of subcutaneous and orthotopic PDX tumors (Fig. 4). The PDX tumors retained key features of primary GBM, including high cellular density, nuclear atypia, pleomorphism, high mitotic activity, geographic necrosis, and an infiltrative growth pattern (Fig. 4**A**). Notably, both subcutaneous and intracranial PDX tumors showed increased cellular compactness compared to their corresponding primary tumors across all six examined GBM samples. Orthotopic PDX tumors further demonstrated typical features associated with astrocytic differentiation, including nuclear atypia, cellular pleomorphism, prominent cytoplasmic processes, and an infiltrative growth pattern. Interestingly, xenografts from GBM3 (P1 and P2) revealed notable cellular dedifferentiation characterized by acquisition of primitive embryonal features, including marked nuclear hyperchromasia, significantly elevated nuclear-to-cytoplasmic ratios, and cytoplasmic scarcity, which were not observed in the primary tumor specimen (P0). This phenotypic shift suggests potential evolution of tumor cell characteristics during serial passaging in the xenograft model. In contrast, IHC analysis showed that the expression patterns of multiple markers were consistently recapitulated in both subcutaneous and orthotopic PDX models, despite inter-patient heterogeneity of staining intensity and the proportion of immunopositive cells, particularly in P53 and GFAP expression profiles (Fig. 4**B**). Additionally, Ki-67 labeling indices indicated comparable proliferative activity between primary GBM specimens and their corresponding PDX tumors, further supporting the phenotypic fidelity of the xenograft models. However, neither the original patient tumors nor the derived subcutaneous or intracranial PDX tumors showed detectable expression of chromogranin, synaptophysin, or nestin, indicating the absence of neuronal, neuroendocrine, and stem/progenitor cell features (**Additional file 1: Figure S2**).

### Detection of highly amplified ecDNA-driven MYC(N) oncogene in PDX tumors

We further investigated comprehensive molecular characteristics of the PDX models in two samples, GBM1 and GBM3, based on multi-omics analysis including WES, RNA-seq, and DNA CpG methylome sequencing (Fig. 1**B**). Among the multiple PDXs generated from GBM1 (Fig. 2**C**), we selected a recurrent subcutaneous PDX tumor from S2 (designated S2_R) and an intracranial tumor from S3 (designated B3) for comprehensive sequencing analysis (Fig. 1**B** and **Additional file 1: Table S1**). The S2_R tissue was collected from a tumor that recurred after surgical excision of the initial subcutaneous PDX tumor (S2) from the engrafted mouse.

**Fig. 5:**
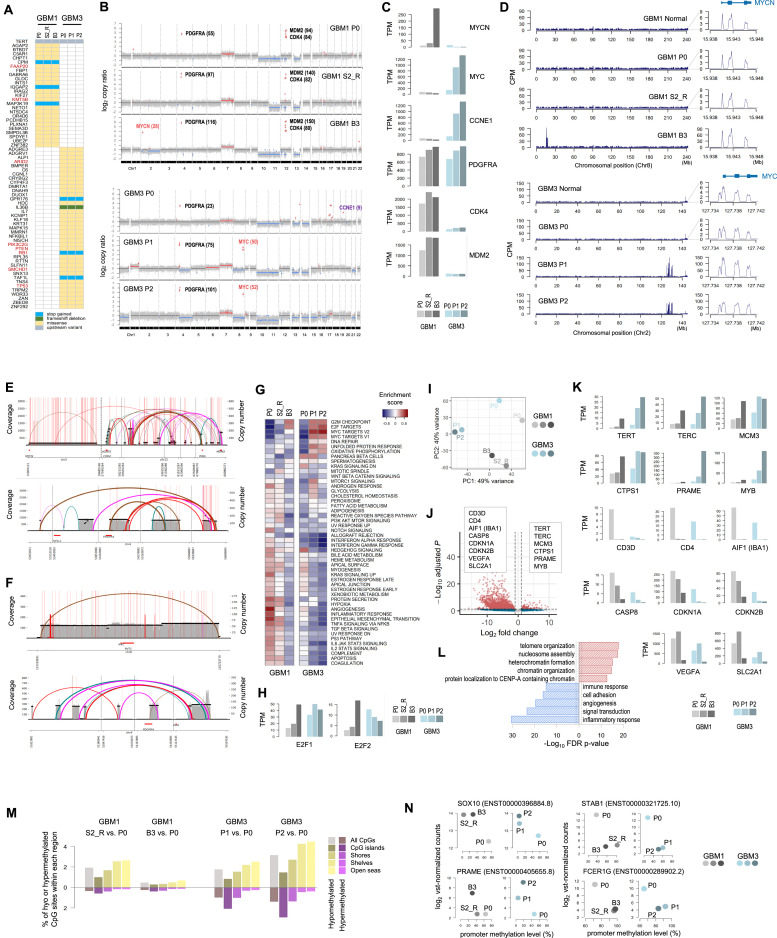
Comprehensive genomic, transcriptomic, and epigenomic analysis of patient tumors and PDX tumors in GBM1 and GBM3. (A) Nonsynonymous mutations preserved in PDX tumors. (B) Copy number variation (CNV) analysis of patient and PDX tumors. (C) Expression levels of MYC(N), CCNE1, PDGFRA, CDK4, and MDM2 presented as transcripts per kilobase million (TPM). (D) Increased counts per million (CPM) of mapped reads in MYCN or MYC region. (E) Amplicon structures detected in GBM1 by the AmpliconSuite-pipeline. (F) Amplicon structures detected in GBM3 by the AmpliconSuite-pipeline. (G) Gene Set Variation Analysis (GSVA) analysis with variance stabilizing transformation (vst)-normalized gene expression profiles. (H) Expression levels of E2F1 and E2F2. (I) Principal component analysis (PCA) based on vst-normalized gene expression profiles. (J) Volcano plot showing differentially expressed genes (DEGs) between intracranial PDXs and patient tumors. (K) TPM values of representative DEGs. (L) Gene set enrichment analysis with DEGs based on DAVID Bioinformatics Resources (red: up-regulated DEGs; blue: down-regulated DEGs). (M) Percentages of hypo- or hyper-methylated CpG sites across different genomic regions. (N) Representative canonical transcripts showing the negative correlation between expression and methylation levels.

As evidenced in previous studies [3,6,39], nearly all mutations identified in the primary tumors (P0) were retained in both subcutaneous and intracranial PDXs, including mutations in well-known GBM tumorigenesis-related genes, including the TERT promoter and tumor suppressors such as RB, TP53, and PTEN (Fig. 5**A** and **Additional file 1: Table S2**). In CNV analysis, we detected several focal amplifications that were either newly detected or lost in subcutaneous and/or intracranial PDX tumors ( Fig. 5**B**), which were corroborated by gene expression profiles (Fig. 5**C**). More specifically, in GBM1, focal amplification of MYCN was identified in the intracranial PDX tumor (B3), but not in the recurrent subcutaneous PDX (S2_R) or the primary tumor (P0). Notably, the multiple subcutaneous PDX models (S1-S4) from GBM1 exhibited substantial differences in growth rates (Fig. 2**C**), suggesting that distinct subpopulations of tumor cells were expanded in each model. The fastest growth rate was observed in S3, while S2 showed much slower growth, which is consistent with the focal amplification of MYCN observed in B3 derived from S3. In GBM3, focal amplification of MYC was observed in both subcutaneous (P1) and intracranial (P2) PDXs, but not in the primary tumor (P0). However, the MYC amplification was initially detected with a relatively low copy number (5) in the primary tumor (P0) of GBM3 by targeted sequencing (Fig. 1**C**). In search of the MYCN or MYC amplification in the primary tumors, we calculated counts per million (CPM) mapped reads in WES data. However, no clear evidence was observed in the primary tumors (Fig. 5**D**), suggesting that only a very small subpopulation of the primary tumor harbored the MYC(N) amplification, and the detection of MYC copy number gain in Fig. 1**C** was the result of serendipitous capturing of the rare subpopulation in the GBM3 primary tumor. Considering the histological transformation observed in GBM3 PDX tumors, which displayed dedifferentiated primitive embryonal features (Fig. 4**A**), the morphological alterations were likely driven by enhanced amplification of MYC. On the other hand, amplification of CCNE1 present in the primary GBM3 tumor was lost in PDXs, while PDGFRA, CDK4, and MDM2 amplifications were preserved from primary tumors to all PDX tumors (Fig. 5**B**). Given that ecDNA is one of the major mechanisms of focal amplification of oncogenes in GBM [25,40], we applied GCAP [29], a computational tool for detecting ecDNA using WES data. Indeed, many genes within focally amplified regions were classified as ecDNA cargo genes, including CDK4, MDM2, PDGFRA, and MYC (**Additional file 1: Table S3**). To further validate ecDNA presence and to reconstruct the structure of focally amplified regions, we performed WGS on two intracranial PDX tumors: B3 from GBM1 and P2 from GBM3. As expected, the presence of ecDNAs was predicted in both PDX tumors, three ecDNAs in GMB1 and two ecDNAs in GBM3 (**Additional file 1: Table S4-S6**). In the B3 of GBM1, two ecDNAs in amplicon 1 harbored MYCN and CDK4/MDM2, respectively, while one ecDNA in amplicon 2 contained PDGFRA. Similarly, the P2 PDX tumor from GBM3 harbored two ecDNAs, one ecDNA containing MYC and the other with PDGFRA (Fig. 5**E and** 5**F**). Congruently, GSVA analysis with gene expression profiles revealed markedly increased enrichment of MYC target gene sets in GBM3 PDXs, P1 and P2 (Fig. 5**G**). In B3 of GBM1, considerable upregulation of E2F1 and E2F2 was observed (Fig. 5H), along with enhanced enrichment of the E2F target gene set (Fig. 5**G**), consistent with a recent study that reported increased E2F expression induced by MYCN amplification [41].

Principal component analysis (PCA) based on gene expression profiles revealed that PDX tumors clustered more closely according to their origin (Fig. 5**I**). Differential expression analysis identified 2,373 DEGs between intracranial PDXs and primary tumors (P0), including 277 upregulated and 2,096 downregulated genes in P2 (absolute log_2_ fold change ≥ 1, FDR *p*-value < 0.05) (Fig. 5**J and** 5**K**). Up-regulated genes included TERT and TERC (telomerase components), MCM3 (a replication marker), CTPS1 (a poor prognosis biomarker in various cancers), PRAME (a well-known tumor antigen), and MYB (an oncogene), while down-regulated genes included immune cell markers (CD3D, CD4, AIF1), an apoptosis-related gene (CASP8), tumor suppressors (CDKN1A, CDKN2B), and angiogenesis-associated genes (VEGFA, SLC2A1). Gene set enrichment analysis also confirmed that upregulated DEGs were associated with cell proliferation and cell cycle, while downregulated DEGs were enriched for immune response and angiogenesis-related genes (Fig. 5**L**). Consistent with previous studies [9,39,42], immune-related genes were profoundly down-regulated during the generation of subcutaneous PDXs. In contrast, angiogenesis or hypoxia-related genes were specifically down-regulated in intracranial PDXs but not in subcutaneous models (Fig. 5**K**), which was further supported by GSVA results (Fig. 5**G**).

Subsequently, we investigated changes in DNA CpG methylation profiles in PDX models. Among a total of 1,526,644 CpG sites (depth ≥ 10), < 5 % were differentially methylated in PDXs compared to the corresponding primary tumors (Fig. 5M). In general, we observed that hypomethylation occurred dominantly in PDXs, more frequently in CpG shelves and open seas. In contrast, hypermethylation occurred mainly in CpG islands and shores. In GBM3, the extent of DNA methylation changes was higher in the intracranial PDX compared to the subcutaneous PDX across all genomic regions, consistent with a previous report [43]. In GBM1, the extent of methylation changes in S2_R was comparable to that observed in P1 of GBM3, whereas only minimal alterations were detected in the intracranial PDX (B3), highlighting distinct epigenetic—as well as genetic—differences between S2_R and B3.

Finally, we examined correlations between promoter methylation and expression of canonical transcripts and identified negative correlations in several genes, including cancer-related (SOX10, PRAME) or immune-related genes (STAB1, FCER1G) (Fig. 5N).

### Potential therapeutic effects of adoptively transferred NK cells in an orthotopic PDX model harboring ecDNA-driven MYC and PDGFRA amplification

**Fig. 6:**
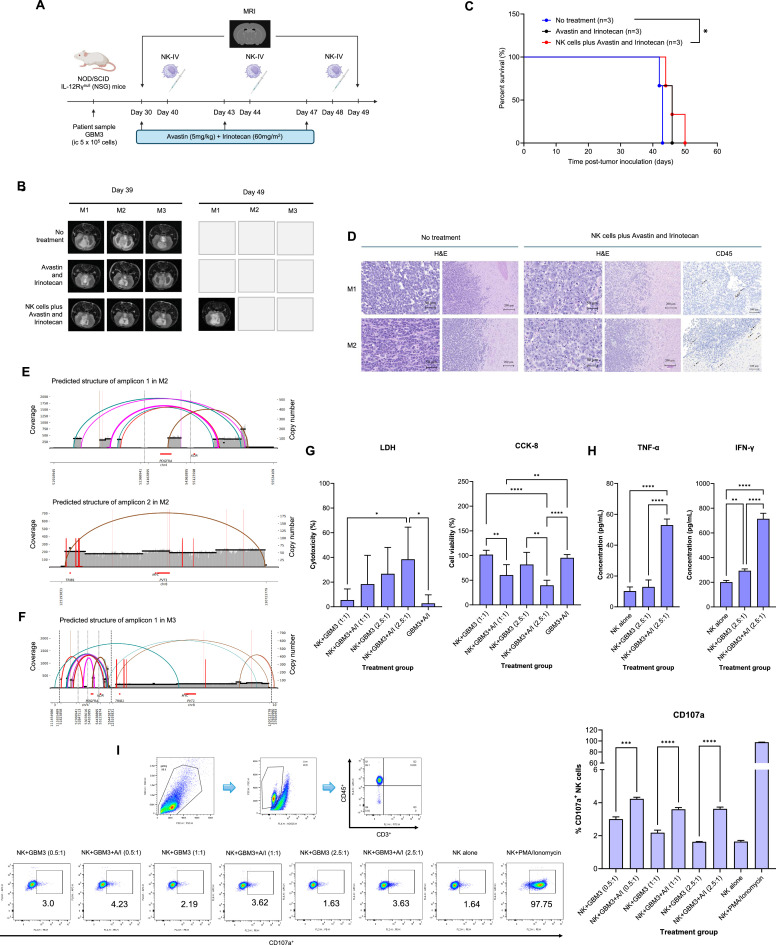
Therapeutic effects of NK cells in combination with Avastin® and irinotecan in vitro and in an orthotopic intracranial PDX model of GBM3. (A) Schematic overview of the experimental treatment protocol. (B) Tumor volumes in PDX-bearing mice before and after treatment, assessed by T2-weighted MRI. (C) Kaplan–Meier survival curves of different treatment groups. (D) Histological features in PDX tumors with or without treatment, and NK cell infiltration visualized by CD45 immunostaining. (E, F) Validation of amplified ecDNAs carrying MYC and PDGFRA oncogenes in intracranial PDX tumors after combined treatment (NK cells with Avastin® and irinotecan) using whole-genome sequencing (WGS). (G) Cytotoxic activity of NK cells against primary GBM3 cells with or without Avastin® plus irinotecan pretreatment. (H) IFN-γ and TNF-α secretion by NK cells after co-culture with primary GBM3 cells, with or without Avastin® plus irinotecan pretreatment. (I) Degranulation activity of expanded NK cells against primary GBM3 cells with or without Avastin® plus irinotecan pretreatment. NK-IV: intravenously injected NK cells; M1, M2, M3: individual mouse identification numbers; 0.5:1, 1:1, and 2.5:1: effector-to-target (E:T) ratios of NK cells to tumor cells; A/I: Avastin® plus irinotecan; PMA/Ionomycin: positive control. ****: p-value < 0.0001; ***: p-value < 0.001; **: p-value < 0.01; *: p-value < 0.05; Figure 6A was created with BioRender (http://biorender.com/).

To assess the therapeutic potential of immunotherapy in GBM, the effect of adoptively transferred NK cells combined with standard second-line GBM treatment (Avastin® plus irinotecan) was evaluated in an orthotopic intracranial PDX model from GBM3, which harbored ecDNA-driven amplification of MYC and PDGFRA. Mice were divided into three treatment groups, with three mice per group: no treatment, Avastin® plus irinotecan, and adoptively transferred NK cells plus Avastin® and irinotecan. The treatment regimen and timeline are shown in Fig. 6**A**.

To assess the therapeutic outcomes, intracranial tumors were monitored using T2-weighted MRI before (Day 39) and after treatment (Day 49) (Fig. 6**B**). Survival analysis revealed no significant difference between the no-treatment group and the group treated with Avastin® and Irinotecan alone (Fig. 6**C**), indicating resistance to these treatments in GBM tumors with ecDNA-driven MYC and PDGFRA amplification. However, the combination of NK cells with Avastin® and irinotecan significantly improved survival (log-rank test *p*-value = 0.03), suggesting that the addition of NK cells may enhance therapeutic efficacy in GBM tumors with ecDNA-driven MYC and PDGFRA amplification.

Histopathological assessment showed that PDX tumors in the untreated group retained the invasive characteristics commonly observed in primary GBM tumors (Fig. 6**D**). In contrast, combination therapy with NK cells, Avastin®, and irinotecan demonstrated significant cytoreduction, characterized by marked diminution in tumor cell density. This therapeutic effect was accompanied by a pronounced expansion of the intervening glial stroma, resulting in increased spatial separation between the residual neoplastic cells. The presence of NK cells in the tumors was confirmed by CD45 immunohistochemical staining, which revealed the presence of CD45-positive cells in tumors treated with the combination of adoptively transferred NK cells, Avastin®, and irinotecan. Notably, CDK4 protein expression remained absent in both untreated and combination-treated tumors (**Additional file 1: Figure S3**). However, whole-genome sequencing revealed that ecDNA-driven amplification of MYC and PDGFRA persisted after therapy. Both MYC and PDGFRA remained markedly amplified, indicating that treatment was insufficient to eradicate ecDNA (Fig. 6**E** and 6**F**).

### Cytotoxic effects of expanded NK cells against primary GBM cells used to establish the orthotopic PDX model harboring ecDNA-driven MYC and PDGFRA amplification

To further evaluate the therapeutic potential of immunotherapy, we examined the effect of expanded NK cells in combination with Avastin® and irinotecan using in vitro assays. The primary GBM3 cells used in these experiments were the same patient-derived cells employed to establish the orthotopic PDX model with ecDNA-driven MYC and PDGFRA amplification.

In the short term (5 hours), NK cells combined with Avastin® and irinotecan exhibited significantly greater cytotoxicity against primary GBM3 cells compared with Avastin® plus irinotecan alone (*p*-value = 0.025). Degranulation of CD107a⁺ NK cells was also significantly enhanced when co-cultured with primary GBM3 cells pretreated with Avastin® plus irinotecan relative to untreated primary GBM3 cells (*p*-value = 0.0001 at 0.5:1 E:T; *p*-value < 0.0001 at 1:1 and 2.5:1 E:T ratios) (Fig. 6**G** and 6**I**).

In the long term (24 hours), NK cells combined with Avastin® and irinotecan further enhanced cytotoxicity compared with GBM3 plus Avastin® plus irinotecan alone (*p*-value = 0.0033 at 1:1 E:T) and compared with NK cells co-cultured with untreated primary GBM3 cells (*p*-value = 0.0035 at 1:1 E:T). At the 2.5:1 ratio, NK cells plus Avastin® and irinotecan also demonstrated significantly more potent cytotoxicity than either Avastin® plus irinotecan alone (*p*-value < 0.0001) or NK cells against untreated primary GBM3 cells (*p*-value = 0.0029) (Fig. 6**G**).

Regarding cytokine production (Fig. 6**H**), at the 2.5:1 E:T ratio, TNF-α secretion was not significantly increased when NK cells were cultured with primary GBM3 cells compared with NK cells alone, whereas IFN-γ secretion was significantly enhanced (*p*-value = 0.0034). Moreover, NK cells co-cultured with primary GBM3 cells pretreated with Avastin® and irinotecan secreted significantly higher levels of both TNF-α and IFN-γ compared with NK cells alone (*p*-value < 0.0001 for both) and compared with NK cells cultured with untreated primary GBM3 cells (*p*-value < 0.0001 for both).

## Discussion

PDX models are invaluable for recapitulating GBM biology and testing therapies in clinically relevant settings. Here, we established orthotopic intracranial PDXs via subcutaneous expansion from fresh or cryopreserved tumors. Subcutaneous engraftment succeeded in 50 % of fresh and 85.7 % of cryopreserved samples, although prolonged cryopreservation delayed growth. A higher Ki-67 index in donor tumors correlated with faster PDX growth, highlighting the influence of proliferative activity. Once established, orthotopic tumors engrafted with 100 % success.

Sex and age are recognized as important modifiers of GBM biology. Women with GBM generally show more prolonged survival than men up to 60 years of age, but this benefit appears to diminish after menopause, likely in relation to estradiol loss [[44], [45], [46]]. In our cohort, all female donors were older than 60, and PDX engraftment rates were observed to be higher from male donors. While sex-linked differences in tumor stemness and the extent of necrosis have been reported, the available evidence is insufficient to ascribe our engraftment pattern to a specific mechanism; these findings can be regarded as a hypothesis-generating. Using exclusively female SCID hosts is unlikely to have biased the results, consistent with prior studies [47].

Intracranial PDXs reproduced hallmark GBM features, including perivascular invasion. Prior work has shown that invasion patterns vary with implantation method, with flank models often selecting for perivascular growth [[48], [49], [50]]. In our study, most tumors showed diffuse infiltration near the injection site, while one model spread to distant regions such as the corpus callosum and cerebellum. A recent study reported that reduced lamin A/C enhances nuclear flexibility, facilitating penetration of tumor cells into surrounding tissue and promoting long-distance migration along white matter tracts [15]. However, subtle factors such as positioning, needle angle, or backflow might have influenced tumor growth location, although the stereotactic infusion pump minimized injection variability. Validation in larger cohorts will be necessary to confirm reproducibility and reduce variability introduced by the injection technique.

ecDNA plays a critical role in tumor dynamics, promoting the survival of oncogene-bearing cells through unbalanced segregation [[16], [17], [18],^20^,^25^,^51^]. In GBM, ecDNA frequently carries oncogenes such as MYC(N), EGFR, PDGFRA, MET, the MECOM/PIK3CA/SOX2 cluster, and the CDK4/MDM2 cluster [19,20]. Highly amplified ecDNA-driven oncogenes have been detected in recurrent GBM tumors as well as derived models, including brain tumor–initiating cell (BTIC) cultures and PDXs [25]. In this study, we identified ecDNA-driven amplifications of MYC(N), PDGFRA, CDK4, and MDM2 in PDX tumors from two patients. While PDGFRA, CDK4, and MDM2 were already prevalent in primary tumors, MYC(N) amplification was not detected or minimally detected, suggesting selective expansion during PDX propagation. Notably, we observed that MYC amplification was linked to embryonal-like histology in GBM3, whereas the fastest-growing PDX in GBM1 contained MYCN amplification, while a non-amplified counterpart grew 24-fold more slowly. These observations underscore the capacity of ecDNA-bearing subclones to undergo rapid clonal expansion and profoundly alter tumor growth dynamics. However, because our analyses were limited to two PDX models, larger cohorts will be necessary to validate these findings and better define molecular changes across P0, P1, and P2 passages.

Functionally, MYC(N) regulates cell cycle and metabolism, supporting glioma stem cell proliferation. Their ecDNA-driven amplification promotes self-renewal but often cooperates with other oncogenes and tumor suppressor loss. PDGFRA, the second most frequently altered receptor tyrosine kinase (RTK) in IDH-wildtype GBM after EGFR, is also ecDNA-amplified and drives invasion, motility, and therapy resistance [[52], [53], [54], [55]]. The concurrent amplification of MYC and PDGFRA observed in our models likely sustains both stemness and aggressiveness. Moreover, ecDNA has been linked to immune evasion through reduced immune infiltration and downregulation of antigen presentation [56], further highlighting the need for combination approaches. Guided by these insights, we evaluated NK cell therapy combined with Avastin® and irinotecan in both primary GBM cells and their orthotopic PDX models harboring ecDNA-driven MYC and PDGFRA amplifications.

In primary GBM3 cells, the combination of Avastin® plus irinotecan with NK cells enhanced NK cell function compared to single treatments, accompanied by increased degranulation and elevated secretion of IFN-γ and TNF-α. This combination also showed synergistic effects in orthotopic PDX models harboring ecDNA-driven MYC and PDGFRA amplifications. However, given the limited sample size, these findings require confirmation in larger cohorts. WGS confirmed that MYC- and PDGFRA-driven ecDNA persisted after therapy, reflecting their stability.

A limitation of our study is that we did not perform direct visualization by fluorescence in situ hybridization (FISH) to confirm the presence of ecDNA. Although WGS provided strong evidence for ecDNA structures, complementary validation with FISH would have further substantiated our findings. Likewise, another limitation is that, apart from CDK4, we did not examine additional markers such as cyclin E, Ki-67, or caspase-3, nor perform histological evaluation of the Avastin®/irinotecan–only group. Including these analyses would have provided stronger comparative support. Although NSG mice lack functional T, B, and NK cells, residual innate immune components such as microglia and neutrophils remain and could influence interactions with adoptively transferred allogeneic NK cells. Future studies with larger cohorts should investigate how combination therapy modulates these residual immune populations in immunodeficient mice, and further assess its effects in humanized models that more accurately recapitulate the human immune microenvironment.

## Conclusion

This study identifies ecDNA as a critical driver of GBM progression through oncogene amplification and persistence after treatment. Orthotopic intracranial PDX models provided a clinically relevant platform, where NK cell therapy combined with Avastin® and irinotecan showed activity against ecDNA-driven tumors. These findings highlight the need for future strategies that integrate immunotherapy with approaches directly targeting ecDNA to achieve durable clinical benefit in GBM.

## Ethics approval and consent to participate

All procedures followed ethical guidelines and regulations approved by the Institutional Review Board at the Chonnam National University Hwasun Hospital (CNUHH-2022-144), with informed consent obtained from all patients before surgery.

## Consent for publication

Not applicable.

## Availability of data and materials

The datasets analyzed in the current study have been deposited into Sequence Read Archive (SRA) database under accession number PRJNA1261796

## Funding

This work was supported by Industry-university Cooperation Collabo R&D Program funded by the Ministry of SMEs and Startups, Republic of Korea (RS-2023-00226999), by a grant (grant no HCRI20004, HCRI22004) from the Chonnam National University Hwasun Hospital Institute for Biomedical Science, Republic of Korea, and by a National Research Foundation (NRF) grant of Korea funded by the Ministry of Science & ICT (2020R1I1A3073845, RS-2024-00353589, RS-2024-00406625).

## CRediT authorship contribution statement

**Thi-Anh-Thuy Tran:** Writing – review & editing, Writing – original draft, Visualization, Methodology, Investigation. **Sinae An:** Writing – review & editing, Writing – original draft, Supervision, Methodology, Investigation, Funding acquisition, Formal analysis. **Junghyun Lim:** Writing – review & editing, Writing – original draft, Supervision, Project administration, Funding acquisition. **Young-Hee Kim:** Investigation. **Ahyeon Shim:** Writing – review & editing, Software, Formal analysis. **Taewoo Han:** Writing – review & editing, Software, Formal analysis. **Hawsan Kim:** Writing – review & editing, Formal analysis. **Sue-Jee Park:** Writing – review & editing. **Yeong Jin Kim:** Writing – review & editing. **Kyung-Sub Moon:** Writing – review & editing. **In-Young Kim:** Writing – review & editing. **Shin Jung:** Writing – review & editing. **Chul Won Lee:** Writing – review & editing. **Kyung-Hwa Lee:** Writing – review & editing, Writing – original draft, Supervision. **Ae Kyung Park:** Writing – review & editing, Writing – original draft, Supervision, Project administration, Funding acquisition. **Tae-Young Jung:** Writing – review & editing, Writing – original draft, Supervision, Resources, Project administration, Funding acquisition, Conceptualization.

## Declaration of competing interest

The authors declare that they have no known competing financial interests or personal relationships that could have appeared to influence the work reported in this paper.

## Appendix Supplementary materials

Image, application 1
