# Supplementary material for: Generation of orthotopic intracranial glioblastoma patient-derived xenograft models: insights into extrachromosomal DNA-driven MYC(N) and PDGFRA oncogene amplification and preliminary therapeutic evaluation
Source: Neoplasia. 2025 Sep 25;69:101233. doi: 10.1016/j.neo.2025.101233 (PMC12505004; doi:10.1016/j.neo.2025.101233)
Supplement: Supplementary file 1 [file mmc1.docx]

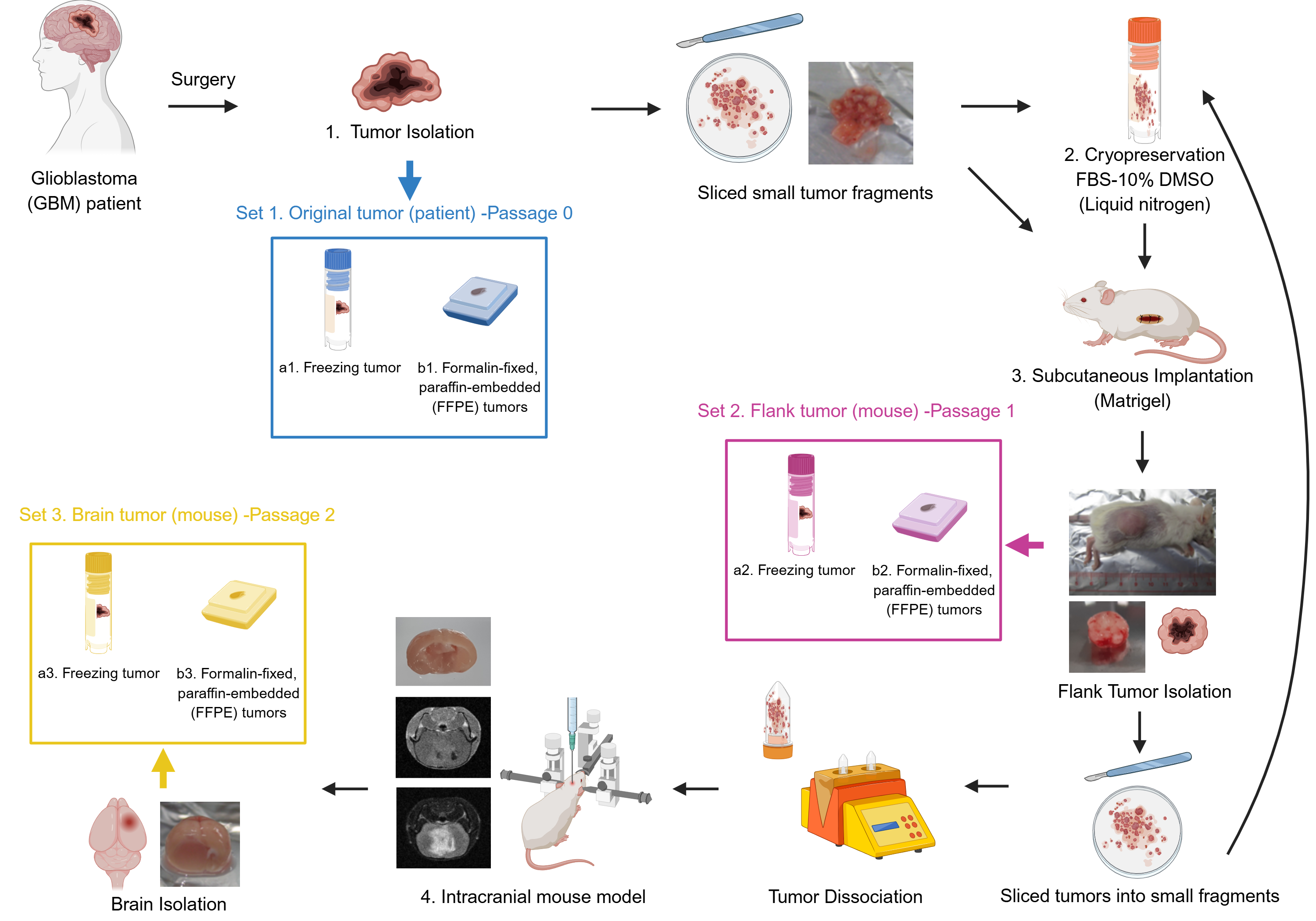


**Additional File 1: Figure S1. General protocol for generating an orthotopic intracranial PDX mouse model.** Orthotopic intracranial PDX models were established from heterotopic subcutaneous PDX models derived from fresh or cryopreserved GBM patient tumor tissues. Figure was created using Biorender (http://biorender.com/).


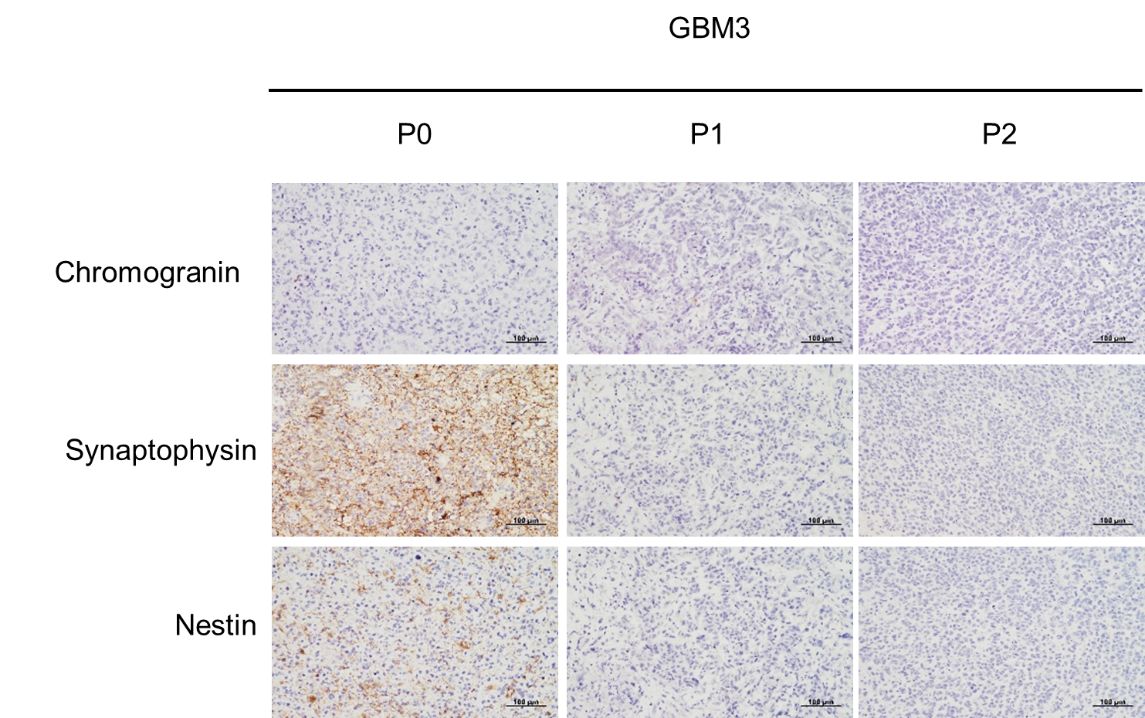


**Additional file 1: Figure S2. Histopathological comparison of patient tumors with PDX tumors in subcutaneous and intracranial PDX mouse models. Immunohistochemical (IHC) staining for chromogranin, synaptophysin, nestin, and MYC.**


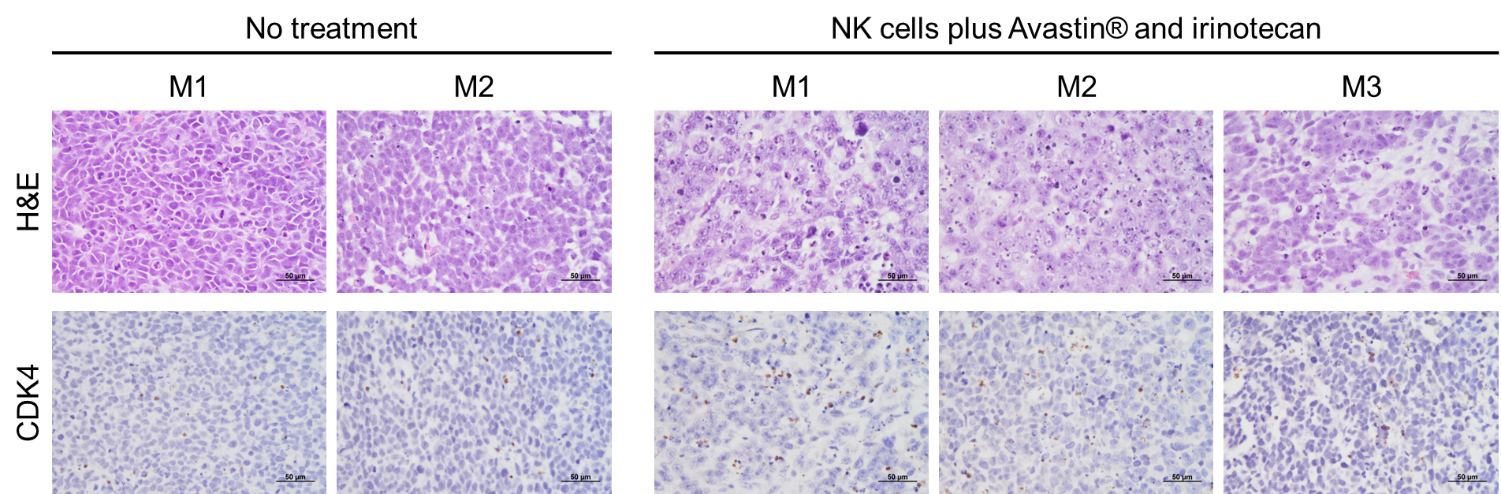


**Additional file 1: Figure S3. Histology and CDK4 expression in PDX tumors following treatment with NK cells plus Avastin® and irinotecan or without treatment.**


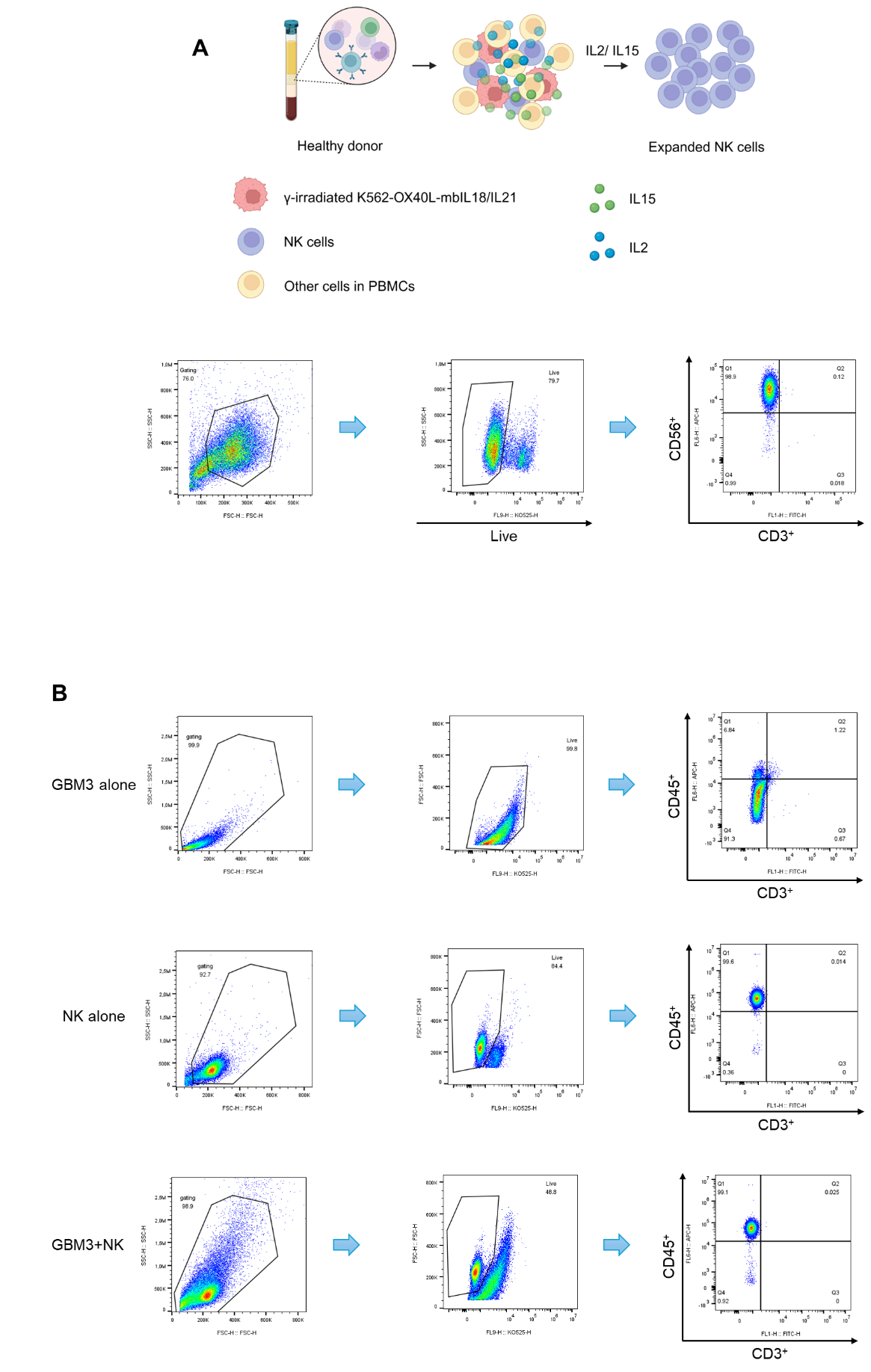


**Additional file 1: Figure S4. Gating strategy.** (A) Schematic overview of primary peripheral NK cell expansion using modified K562-OX40L-mbIL18/IL21 feeder cells, and gating strategy for assessing NK cell purity. (B) Gating strategy for detection of CD107a⁺ NK cells after co-culture with primary GBM3 cells. Figure S4A was created with BioRender (<http://biorender.com/>).

**Additional file 1: Table S1. Detailed information on subcutaneous and intracranial PDX models generated from 16 GBM patient tumors**

| **Patient**  **ID** | **Cryopreservation duration (week)** | **Heterotopic Subcutaneous (SC) PDX model** | | | | | | **Orthotopic Intracranial (IC) PDX model** | | | | | | |
| --- | --- | --- | --- | --- | --- | --- | --- | --- | --- | --- | --- | --- | --- | --- |
|  |  | **Matrigel use** | **Tumor**  **formation** | **Expansion duration (week)** | **Tumor volume (mm^3^)** | **PDX tumor for IC model label** | **Recurrent tumor label** | **Generating  IC model** | **Mouse No.** | **Engrafted mouse type** | **Tumor formation** | **First MRI-detected tumor (week)** | **PDX**  **tumor label** | **Treatment** |
| GBM1 | 0W | No | Yes | 9 | 528.8 | S1 |  | Yes | No.1 | NSG | Yes | ~~-~~ | B1/2 |  |
|  |  | Yes | Yes | 9 | 118.1 | S2 | S2_R* |  |  |  |  |  |  |  |
|  |  | Yes | Yes | 9 | 911.6 | S3 |  | Yes | No.1 | Nude | Yes | ~~-~~ | B3* |  |
|  |  |  |  |  |  |  |  |  | No.2 | Nude | Yes | ~~-~~ |  |  |
|  |  | No | Yes | 9 | 37.9 | S4 |  | Yes | No.1 | NSG | Yes | 4 | B4 |  |
|  |  |  |  |  |  |  |  |  | No.2 | NSG | No |  |  |  |
| GBM2 | 1W | Yes | Yes | 13 | 929.67 |  |  | No |  |  |  |  |  |  |
| GBM3 | 3W | Yes | Yes | 11 | 1691.2 | P1* |  | Yes | No.1-3 | NSG | Yes | ~~-~~ | P2* | No treatment |
|  |  |  |  |  |  |  |  |  | No.4-6 | NSG |  | ~~-~~ |  | Avastin+Irinotecan |
|  |  |  |  |  |  |  |  |  | No.7-9 | NSG |  | ~~-~~ |  | Avastin+Irinotecan+NK cells |
| GBM4 | 10W | Yes | Yes | 19 | 1665.77 |  |  | No |  |  |  |  |  |  |
|  | 12W | Yes | Yes | 19 | 1248.56 |  |  | No |  |  |  |  |  |  |
|  | 26W | Yes | Yes | 26 | 1121.55 |  |  | No |  |  |  |  |  |  |
| GBM5 | 8W | Yes | Yes | 17 | 1649.5 |  |  | No |  |  |  |  |  |  |
| GBM6 | 17W | Yes | Yes | 14 | 852.08 | P1 |  | Yes | No.1 | NSG | Yes | 9 | P2 |  |
|  |  |  |  |  |  |  |  |  | No.2 | NSG | Yes | 6 |  |  |
|  |  |  |  |  |  |  |  |  | No.3 | NSG | Yes | 9 |  |  |
|  | 39W | Yes | Yes | 11 | 1466.89 |  |  | No |  |  |  |  |  |  |
| GBM7 | 21W | Yes | Yes | 9 | 1646.51 | P1 |  | Yes | No.1 | NSG | Yes | 6 | P2 |  |
|  |  |  |  |  |  |  |  |  | No.2 | NSG | Yes | 8 |  |  |
| GBM8 | 39W | Yes | Yes | 23 | 1224.79 |  |  | No |  |  |  |  |  |  |
| GBM9 | 48W | Yes | Yes | 31 | 1007.13 |  |  | No |  |  |  |  |  |  |
| GBM10 | 53W | Yes | Yes | 27 | 1572.43 |  |  | No |  |  |  |  |  |  |
| GBM11 | 55W | Yes | Yes | 23 | 838.56 |  |  | No |  |  |  |  |  |  |
|  | 56W | Yes | Yes | 30 | 1117.5 |  |  | No |  |  |  |  |  |  |
| GBM12 | 56W | Yes | Yes | 13 | 1481.81 | P1 |  | Yes | No.1 | NSG | Yes | 11 | P2 |  |
|  |  |  |  |  |  |  |  |  | No.2 | NSG | Yes | 14 |  |  |
|  |  |  |  |  |  |  |  |  | No.3 | NSG | Yes | 12 |  |  |
| GBM13 | 63W | Yes | Yes | 25 | 1174.96 | P1 |  | Yes | No.1 | NSG | Yes | 10 | P2 |  |
|  |  |  |  |  |  |  |  |  | No.2 | NSG | Yes | 9 |  |  |
| GBM14 | 0W | Yes | No |  |  |  |  |  |  |  |  |  |  |  |
| GBM15 | 15W | Yes | No |  |  |  |  |  |  |  |  |  |  |  |
| GBM16 | 29W | Yes | No |  |  |  |  |  |  |  |  |  |  |  |

* Comprehensive genomic, transcriptomic, epigenomic analysis with next-generation sequencing (NGS)

**Additional file 1: Table S2.** **Somatic mutations detected by whole exome sequencing (WES) in patient tumors, subcutaneous PDXs, and intracranial PDXs from GMB1 and GBM3**

| **CHR** | **POS** | **REF** | **ALT** | **Gene Symbol** | **cDNA Change** | **Variant Classification** | **Tumor tissue with mutation** | | | | | |
| --- | --- | --- | --- | --- | --- | --- | --- | --- | --- | --- | --- | --- |
|  |  |  |  |  |  |  | **GBM1**  **(P0)** | **GBM1**  **(S2_R)** | **GBM1**  **(B3)** | **GBM3**  **(P0)** | **GBM3**  **(P1)** | **GBM3**  **(P2)** |
| chr5 | 1295113 | G | A | TERT |  | FIVE_PRIME_FLANK | Yes | Yes | Yes | Yes | Yes | Yes |
| chr1 | 46859640 | AGTT | A | CYP4Z2P | c.e9-14AACT>T | RNA | Yes | Yes | Yes | Yes | - | - |
| chr1 | 2185483 | G | A | FAAP20 | c.365C>T | MISSENSE | Yes | Yes | Yes | - | - | - |
| chr1 | 2594731 | C | T | MMEL1 | c.e17-59G>A | INTRON | Yes | Yes | Yes | - | - | - |
| chr1 | 6466438 | A | G | TNFRSF25 |  | FIVE_PRIME_FLANK | Yes | Yes | Yes | - | - | - |
| chr1 | 16758502 | G | A | ENSG00000290851 | c.e11+36C>T | RNA | Yes | Yes | Yes | - | - | - |
| chr1 | 17380031 | C | T | PADI6 | c.e4+44C>T | INTRON | Yes | Yes | Yes | - | - | - |
| chr1 | 27955709 | C | T | SMPDL3B | c.716C>T | MISSENSE | Yes | Yes | Yes | - | - | - |
| chr1 | 155051341 | G | A | ADAM15 |  | FIVE_PRIME_UTR | Yes | Yes | Yes | - | - | - |
| chr1 | 168729241 | C | T | DPT |  | FIVE_PRIME_FLANK | Yes | Yes | Yes | - | - | - |
| chr1 | 196317128 | C | T | KCNT2 | c.e21+1102G>A | INTRON | Yes | Yes | Yes | - | - | - |
| chr1 | 201200375 | G | A | IGFN1 | c.597G>A | SILENT | Yes | Yes | Yes | - | - | - |
| chr1 | 204871101 | G | A | NFASC | c.e1+42319G>A | INTRON | Yes | Yes | Yes | - | - | - |
| chr1 | 220627246 | C | T | MARK1 | c.e10-3789C>T | INTRON | Yes | Yes | Yes | - | - | - |
| chr1 | 248388109 | G | A | OR2T6 | c.501G>A | SILENT | Yes | Yes | Yes | - | - | - |
| chr11 | 14254749 | G | A | SPON1 | c.e8+20G>A | INTRON | Yes | Yes | Yes | - | - | - |
| chr11 | 59457807 | A | G | OR4D6 | c.847A>G | MISSENSE | Yes | Yes | Yes | - | - | - |
| chr11 | 68173900 | A | G | KMT5B | c.344T>C | MISSENSE | Yes | Yes | Yes | - | - | - |
| chr11 | 113397894 | G | A | ANKK1 | c.e7-86G>A | INTRON | Yes | Yes | Yes | - | - | - |
| chr12 | 6951960 | C | T | PTPN6 | c.e3-23C>T | INTRON | Yes | Yes | Yes | - | - | - |
| chr12 | 25104408 | G | A | IRAG2 | c.3959G>A | MISSENSE | Yes | Yes | Yes | - | - | - |
| chr12 | 40482994 | G | T | MUC19 | c.10042G>T | RNA | Yes | Yes | Yes | - | - | - |
| chr12 | 57737529 | C | T | AGAP2 | c.718G>A | MISSENSE | Yes | Yes | Yes | - | - | - |
| chr12 | 68859048 | G | A | CPM | c.964C>T | NONSENSE | Yes | Yes | Yes | - | - | - |
| chr12 | 85072868 | G | A | LRRIQ1 | c.e11-39G>A | INTRON | Yes | Yes | Yes | - | - | - |
| chr12 | 101714136 | A | G | CHPT1 | c.320A>G | MISSENSE | Yes | Yes | Yes | - | - | - |
| chr13 | 113096430 | C | T | MCF2L | c.3135C>T | SILENT | Yes | Yes | Yes | - | - | - |
| chr14 | 24574789 | G | A | CTSG | c.225C>T | SILENT | Yes | Yes | Yes | - | - | - |
| chr14 | 50050365 | G | A | LINC01588 | c.e2-2409C>T | RNA | Yes | Yes | Yes | - | - | - |
| chr14 | 59610023 | G | A | RTN1 | c.e2-99C>T | INTRON | Yes | Yes | Yes | - | - | - |
| chr14 | 77694970 | C | T | ALKBH1 | c.e3+70G>A | INTRON | Yes | Yes | Yes | - | - | - |
| chr14 | 93248580 | C | T | BTBD7 | c.2017G>A | MISSENSE | Yes | Yes | Yes | - | - | - |
| chr15 | 21609415 | C | A | LINC02203 |  | FIVE_PRIME_UTR | Yes | Yes | Yes | - | - | - |
| chr15 | 42153191 | G | A | PLA2G4F | c.e6-109C>T | INTRON | Yes | Yes | Yes | - | - | - |
| chr15 | 50596380 | C | T | TRPM7 | c.3165G>A | SPLICE_SITE | Yes | Yes | Yes | - | - | - |
| chr15 | 74295628 | G | A | CCDC33 | c.e11-126G>A | INTRON | Yes | Yes | Yes | - | - | - |
| chr15 | 75738259 | G | A | ENSG00000260288 | c.e1+261G>A | RNA | Yes | Yes | Yes | - | - | - |
| chr15 | 81225408 | G | A | IL16 | c.9G>A | SILENT | Yes | Yes | Yes | - | - | - |
| chr16 | 3675272 | G | A | TRAP1 | c.e8-52C>T | INTRON | Yes | Yes | Yes | - | - | - |
| chr16 | 70474370 | A | G | FCSK | c.e8+31A>G | INTRON | Yes | Yes | Yes | - | - | - |
| chr17 | 8833061 | G | A | PIK3R6 | c.e9+16C>T | INTRON | Yes | Yes | Yes | - | - | - |
| chr17 | 27611456 | C | T | KSR1 | c.e18-38C>T | INTRON | Yes | Yes | Yes | - | - | - |
| chr17 | 48057215 | GC | G | NFE2L1 | c.e4+94GC>G | INTRON | Yes | Yes | Yes | - | - | - |
| chr17 | 56499151 | C | G | ANKFN1 | c.e19+53C>G | INTRON | Yes | Yes | Yes | - | - | - |
| chr17 | 62140397 | C | T | POLRMTP1 | c.e1-242G>A | RNA | Yes | Yes | Yes | - | - | - |
| chr18 | 10762427 | G | A | PIEZO2 | c.e21-73C>T | INTRON | Yes | Yes | Yes | - | - | - |
| chr18 | 49824575 | G | T | MYO5B |  | THREE_PRIME_UTR | Yes | Yes | Yes | - | - | - |
| chr18 | 72749077 | A | G | NETO1 | c.1553T>C | MISSENSE | Yes | Yes | Yes | - | - | - |
| chr19 | 3820064 | CCCAT | C | ZFR2 | c.e11-118ATGGG>G | INTRON | Yes | Yes | Yes | - | - | - |
| chr19 | 14059060 | G | A | PALM3 | c.e2-55C>T | INTRON | Yes | Yes | Yes | - | - | - |
| chr19 | 17365961 | C | T | PLVAP | c.504G>A | SILENT | Yes | Yes | Yes | - | - | - |
| chr19 | 35558679 | C | T | ATP4A | c.1263G>A | SILENT | Yes | Yes | Yes | - | - | - |
| chr19 | 36626898 | G | A | ZNF382 | c.998G>A | MISSENSE | Yes | Yes | Yes | - | - | - |
| chr19 | 47319878 | G | A | C5AR1 | c.101G>A | MISSENSE | Yes | Yes | Yes | - | - | - |
| chr19 | 51235774 | C | T | CD33 | c.e6+98C>T | INTRON | Yes | Yes | Yes | - | - | - |
| chr19 | 54848284 | A | G | KIR3DL2 |  | FIVE_PRIME_FLANK | Yes | Yes | Yes | - | - | - |
| chr19 | 55354558 | C | T | COX6B2 | c.e2+19G>A | INTRON | Yes | Yes | Yes | - | - | - |
| chr2 | 778730 | C | T | Unknown |  | IGR | Yes | Yes | Yes | - | - | - |
| chr2 | 87566608 | T | C | PAFAH1B1P1 | c.e1-156A>G | RNA | Yes | Yes | Yes | - | - | - |
| chr2 | 102239158 | A | G | IL1RL2 | c.e12-34A>G | INTRON | Yes | Yes | Yes | - | - | - |
| chr2 | 106698416 | C | A | EEF1A1P12 | c.e1-260G>T | RNA | Yes | Yes | Yes | - | - | - |
| chr2 | 112725546 | G | A | NT5DC4 | c.1051G>A | MISSENSE | Yes | Yes | Yes | - | - | - |
| chr2 | 121403959 | C | T | CLASP1 | c.e26-412G>A | INTRON | Yes | Yes | Yes | - | - | - |
| chr2 | 134980894 | G | A | MAP3K19 | c.3847C>T | NONSENSE | Yes | Yes | Yes | - | - | - |
| chr2 | 161160685 | C | T | TANK | c.e1+201C>T | INTRON | Yes | Yes | Yes | - | - | - |
| chr2 | 166307222 | C | T | SCN9A | c.e3+148G>A | INTRON | Yes | Yes | Yes | - | - | - |
| chr2 | 219290809 | G | A | PTPRN | c.e20-17C>T | INTRON | Yes | Yes | Yes | - | - | - |
| chr2 | 222201862 | C | T | PAX3 | c.e8-82G>A | INTRON | Yes | Yes | Yes | - | - | - |
| chr2 | 238016631 | G | A | UBE2F | c.280G>A | MISSENSE | Yes | Yes | Yes | - | - | - |
| chr20 | 44258382 | C | T | GDAP1L1 | c.e3-52C>T | INTRON | Yes | Yes | Yes | - | - | - |
| chr20 | 45610063 | G | A | WFDC9 | c.e3-28C>T | INTRON | Yes | Yes | Yes | - | - | - |
| chr21 | 10543278 | C | T | TPTE | c.e7-51C>T | INTRON | Yes | Yes | Yes | - | - | - |
| chr22 | 11318866 | G | A | Unknown |  | IGR | Yes | Yes | Yes | - | - | - |
| chr3 | 32392095 | T | A | CMTM7 | c.e1+30T>A | INTRON | Yes | Yes | Yes | - | - | - |
| chr3 | 37176324 | G | T | LRRFIP2 |  | FIVE_PRIME_UTR | Yes | Yes | Yes | - | - | - |
| chr3 | 77602549 | G | A | ROBO2 | c.e21+58G>A | INTRON | Yes | Yes | Yes | - | - | - |
| chr3 | 98822159 | C | G | DCBLD2 | c.e6-69G>C | INTRON | Yes | Yes | Yes | - | - | - |
| chr3 | 127018429 | G | A | PLXNA1 | c.3796G>A | MISSENSE | Yes | Yes | Yes | - | - | - |
| chr3 | 170654004 | G | A | ENSG00000285218 | c.e3-54040G>A | INTRON | Yes | Yes | Yes | - | - | - |
| chr3 | 184031911 | C | T | HTR3D | c.e1+104C>T | INTRON | Yes | Yes | Yes | - | - | - |
| chr4 | 1172102 | C | T | SPON2 | c.e2+28G>A | INTRON | Yes | Yes | Yes | - | - | - |
| chr4 | 56503388 | G | A | SRP72 |  | THREE_PRIME_UTR | Yes | Yes | Yes | - | - | - |
| chr4 | 146833945 | G | A | TTC29 | c.e9+48C>T | INTRON | Yes | Yes | Yes | - | - | - |
| chr4 | 168246688 | C | A | DDX60 | c.e30+70G>T | INTRON | Yes | Yes | Yes | - | - | - |
| chr5 | 76654215 | C | T | IQGAP2 | c.2194C>T | NONSENSE | Yes | Yes | Yes | - | - | - |
| chr5 | 132657219 | G | A | IL13 |  | FIVE_PRIME_FLANK | Yes | Yes | Yes | - | - | - |
| chr5 | 138253276 | G | A | GFRA3 | c.e7-11C>T | INTRON | Yes | Yes | Yes | - | - | - |
| chr5 | 141157339 | C | T | ENSG00000290893 | c.e1+1343C>T | RNA | Yes | Yes | Yes | - | - | - |
| chr5 | 141245597 | C | T | PCDHB15 | c.19C>T | MISSENSE | Yes | Yes | Yes | - | - | - |
| chr5 | 161686333 | C | T | GABRA6 | c.142C>T | MISSENSE | Yes | Yes | Yes | - | - | - |
| chr5 | 171420098 | G | A | FGF18 |  | FIVE_PRIME_UTR | Yes | Yes | Yes | - | - | - |
| chr5 | 172188429 | C | T | STK10 |  | FIVE_PRIME_FLANK | Yes | Yes | Yes | - | - | - |
| chr5 | 181058529 | C | T | BTNL9 | c.e10+151C>T | INTRON | Yes | Yes | Yes | - | - | - |
| chr6 | 28504261 | G | A | GPX6 |  | THREE_PRIME_UTR | Yes | Yes | Yes | - | - | - |
| chr7 | 1478489 | G | A | INTS1 | c.4507C>T | MISSENSE | Yes | Yes | Yes | - | - | - |
| chr7 | 1620114 | C | T | ENSG00000231476 |  | FIVE_PRIME_FLANK | Yes | Yes | Yes | - | - | - |
| chr7 | 21581811 | G | A | DNAH11 | c.e9-94G>A | INTRON | Yes | Yes | Yes | - | - | - |
| chr7 | 30665249 | C | T | CRHR2 | c.e5+62G>A | INTRON | Yes | Yes | Yes | - | - | - |
| chr7 | 32620464 | C | T | DPY19L1P1 | c.e17-20G>A | RNA | Yes | Yes | Yes | - | - | - |
| chr7 | 44001048 | C | T | SPDYE1 | c.23C>T | MISSENSE | Yes | Yes | Yes | - | - | - |
| chr7 | 44676278 | G | A | OGDH | c.e9+129G>A | INTRON | Yes | Yes | Yes | - | - | - |
| chr7 | 77241956 | A | G | CCDC146 | c.e4+56A>G | INTRON | Yes | Yes | Yes | - | - | - |
| chr7 | 85022397 | C | G | SEMA3D | c.1408G>C | MISSENSE | Yes | Yes | Yes | - | - | - |
| chr8 | 139731957 | C | T | TRAPPC9 | c.e22-22G>A | INTRON | Yes | Yes | Yes | - | - | - |
| chr8 | 143725939 | C | T | FAM83H | c.3522G>A | SILENT | Yes | Yes | Yes | - | - | - |
| chr9 | 6558674 | A | G | GLDC | c.1937T>C | MISSENSE | Yes | Yes | Yes | - | - | - |
| chr9 | 69377524 | C | A | ENTREP1 | c.e5+47C>A | INTRON | Yes | Yes | Yes | - | - | - |
| chr9 | 83683133 | C | T | UBQLN1 | c.e3+67G>A | INTRON | Yes | Yes | Yes | - | - | - |
| chr9 | 83867803 | G | A | KIF27 | c.2815C>T | MISSENSE | Yes | Yes | Yes | - | - | - |
| chr9 | 94605518 | C | T | FBP1 | c.764G>A | MISSENSE | Yes | Yes | Yes | - | - | - |
| chr11 | 27806701 | T | A | CBX3P1 | c.e1+258T>A | RNA | Yes | Yes | - | Yes | - | - |
| chr19 | 32586840 | C | A | PDCD5 | c.e5-18C>A | INTRON | Yes | Yes | - | - | - | - |
| chr10 | 50660017 | A | G | BEND3P1 | c.e1-455A>G | RNA | Yes | - | Yes | - | - | - |
| chr14 | 70247264 | A | G | ADAM21P1 | c.e1-396T>C | RNA | Yes | - | Yes | - | - | - |
| chr6 | 29890779 | A | G | ENSG00000230521 |  | FIVE_PRIME_FLANK | Yes | - | Yes | - | - | - |
| chr14 | 105741430 | G | A | IGHG1 | c.e4-51C>T | RNA | Yes | - | - | - | - | - |
| chr1 | 162760046 | G | A | DDR2 | c.e9+67G>A | INTRON | - | Yes | Yes | - | - | - |
| chr16 | 70829903 | T | C | HYDIN | c.e81+73A>G | INTRON | - | Yes | Yes | - | - | - |
| chr4 | 82938573 | C | T | LIN54 | c.e8+69G>A | INTRON | - | Yes | Yes | - | - | - |
| chr20 | 12380162 | G | A | PA2G4P2 | c.e1+106G>A | RNA | - | Yes | - | - | - | - |
| chr12 | 57200933 | C | A | LRP1 | c.e65-101C>A | INTRON | - | - | Yes | - | - | - |
| chr19 | 8901814 | C | T | MUC16 | c.38716G>A | MISSENSE | - | - | Yes | - | - | - |
| chr19 | 8901815 | A | G | MUC16 | c.38715T>C | SILENT | - | - | Yes | - | - | - |
| chr1 | 19176578 | G | A | UBR4 | c.e20-14C>T | INTRON | - | - | - | Yes | Yes | Yes |
| chr1 | 21847498 | C | T | HSPG2 | c.e62+6G>A | INTRON | - | - | - | Yes | Yes | Yes |
| chr1 | 26345288 | G | A | CRYBG2 | c.2342C>T | MISSENSE | - | - | - | Yes | Yes | Yes |
| chr1 | 44140790 | G | A | KLF18 | c.842C>T | MISSENSE | - | - | - | Yes | Yes | Yes |
| chr1 | 58630930 | G | A | ENSG00000185839 | c.e1+89C>T | RNA | - | - | - | Yes | Yes | Yes |
| chr1 | 169106720 | C | G | ATP1B1 |  | FIVE_PRIME_UTR | - | - | - | Yes | Yes | Yes |
| chr1 | 196273557 | T | A | KCNT2 | c.e25-7303A>T | INTRON | - | - | - | Yes | Yes | Yes |
| chr1 | 228245696 | T | C | OBSCN | c.e13+8T>C | INTRON | - | - | - | Yes | Yes | Yes |
| chr1 | 236208040 | T | TC | GPR137B | c.e7-10T>TC | INTRON | - | - | - | Yes | Yes | Yes |
| chr10 | 5977335 | G | T | IL15RA | c.e1-70C>A | INTRON | - | - | - | Yes | Yes | Yes |
| chr10 | 75030794 | C | T | KAT6B | c.5970C>T | SILENT | - | - | - | Yes | Yes | Yes |
| chr10 | 87933061 | T | C | PTEN | c.302T>C | MISSENSE | - | - | - | Yes | Yes | Yes |
| chr10 | 94720610 | T | C | CYP2C18 | c.e6+73T>C | INTRON | - | - | - | Yes | Yes | Yes |
| chr11 | 30010866 | G | A | KCNA4 | c.1813C>T | SILENT | - | - | - | Yes | Yes | Yes |
| chr12 | 18282722 | C | G | PIK3C2G | c.641C>G | MISSENSE | - | - | - | Yes | Yes | Yes |
| chr12 | 45836622 | C | T | ARID2 | c.739C>T | MISSENSE | - | - | - | Yes | Yes | Yes |
| chr12 | 49129630 | G | A | TUBA1B | c.96C>T | SILENT | - | - | - | Yes | Yes | Yes |
| chr12 | 57806523 | T | C | TSFM | c.e6-1341T>C | INTRON | - | - | - | Yes | Yes | Yes |
| chr12 | 133106160 | C | T | ZNF140 |  | THREE_PRIME_UTR | - | - | - | Yes | Yes | Yes |
| chr13 | 18946945 | A | G | PHF2P2 | c.e20-49T>C | RNA | - | - | - | Yes | Yes | Yes |
| chr13 | 48307369 | T | A | RB1 | c.227T>A | NONSENSE | - | - | - | Yes | Yes | Yes |
| chr13 | 63811546 | G | A | PPP1R2P10 | c.e1-62C>T | RNA | - | - | - | Yes | Yes | Yes |
| chr15 | 39802065 | G | T | GPR176 | c.615C>A | NONSENSE | - | - | - | Yes | Yes | Yes |
| chr15 | 44590630 | A | G | SPG11 | c.e28+1216T>C | INTRON | - | - | - | Yes | Yes | Yes |
| chr15 | 45139547 | C | T | DUOX1 | c.1337C>T | MISSENSE | - | - | - | Yes | Yes | Yes |
| chr15 | 50257511 | C | T | HDC | c.355G>A | MISSENSE | - | - | - | Yes | Yes | Yes |
| chr15 | 57439384 | C | A | CGNL1 | c.1385C>A | MISSENSE | - | - | - | Yes | Yes | Yes |
| chr16 | 11456921 | G | A | ENSG00000188897 | c.3983C>T | MISSENSE | - | - | - | Yes | Yes | Yes |
| chr16 | 19184024 | G | T | SYT17 | c.645G>T | SILENT | - | - | - | Yes | Yes | Yes |
| chr16 | 56904476 | G | A | SLC12A3 | c.e25+14G>A | INTRON | - | - | - | Yes | Yes | Yes |
| chr17 | 7674872 | T | C | TP53 | c.659A>G | MISSENSE | - | - | - | Yes | Yes | Yes |
| chr17 | 7674935 | C | T | TP53 | c.596G>A | MISSENSE | - | - | - | Yes | Yes | Yes |
| chr17 | 11608280 | C | T | DNAH9 | c.569C>T | MISSENSE | - | - | - | Yes | Yes | Yes |
| chr17 | 18148782 | C | T | MYO15A | c.6786C>T | SILENT | - | - | - | Yes | Yes | Yes |
| chr17 | 35353041 | C | T | SLFN11 | c.2021G>A | MISSENSE | - | - | - | Yes | Yes | Yes |
| chr17 | 40496239 | G | A | TNS4 | c.187C>T | MISSENSE | - | - | - | Yes | Yes | Yes |
| chr17 | 41394959 | C | T | KRT31 | c.986G>A | MISSENSE | - | - | - | Yes | Yes | Yes |
| chr17 | 80393331 | C | G | RNF213 | c.e69-14C>G | INTRON | - | - | - | Yes | Yes | Yes |
| chr18 | 2724905 | A | C | SMCHD1 | c.2610A>C | MISSENSE | - | - | - | Yes | Yes | Yes |
| chr18 | 23473649 | C | T | RIOK3 | c.e8+23C>T | INTRON | - | - | - | Yes | Yes | Yes |
| chr18 | 70127673 | C | T | RTTN | c.3212G>A | MISSENSE | - | - | - | Yes | Yes | Yes |
| chr19 | 3150316 | G | A | GNA15 | c.e3+31G>A | INTRON | - | - | - | Yes | Yes | Yes |
| chr19 | 11299001 | C | T | TSPAN16 | c.e2+662C>T | INTRON | - | - | - | Yes | Yes | Yes |
| chr19 | 13277147 | A | C | CACNA1A | c.e23+19T>G | INTRON | - | - | - | Yes | Yes | Yes |
| chr19 | 14647186 | C | T | ADGRE3 | c.232G>A | MISSENSE | - | - | - | Yes | Yes | Yes |
| chr19 | 15641513 | G | A | CYP4F3 | c.98G>A | MISSENSE | - | - | - | Yes | Yes | Yes |
| chr19 | 41018021 | T | A | CYP2B6 |  | THREE_PRIME_UTR | - | - | - | Yes | Yes | Yes |
| chr19 | 46622178 | A | G | PTGIR | c.e3+506T>C | INTRON | - | - | - | Yes | Yes | Yes |
| chr19 | 46730940 | G | A | STRN4 | c.e6+67C>T | INTRON | - | - | - | Yes | Yes | Yes |
| chr19 | 53775594 | G | A | SEPTIN7P8 |  | FIVE_PRIME_FLANK | - | - | - | Yes | Yes | Yes |
| chr19 | 55359173 | G | A | GARIN5B | c.1695C>T | SILENT | - | - | - | Yes | Yes | Yes |
| chr19 | 56622652 | G | T | ZNF71 | c.1545G>T | SILENT | - | - | - | Yes | Yes | Yes |
| chr2 | 88585892 | G | A | EIF2AK3 | c.1599C>T | SILENT | - | - | - | Yes | Yes | Yes |
| chr2 | 113028082 | TC | T | IL36B | c.294delG | FRAME_SHIFT_DEL | - | - | - | Yes | Yes | Yes |
| chr2 | 127719275 | G | A | WDR33 | c.2750C>T | MISSENSE | - | - | - | Yes | Yes | Yes |
| chr2 | 183124361 | C | A | NUP35 | c.e3-3926C>A | INTRON | - | - | - | Yes | Yes | Yes |
| chr2 | 191392245 | G | A | MYO1B | c.e19+44G>A | INTRON | - | - | - | Yes | Yes | Yes |
| chr2 | 208227853 | T | C | Unknown |  | IGR | - | - | - | Yes | Yes | Yes |
| chr2 | 232458718 | G | A | ALPI | c.1270G>A | MISSENSE | - | - | - | Yes | Yes | Yes |
| chr2 | 233759220 | C | T | UGT1A6 | c.e2-7814C>T | INTRON | - | - | - | Yes | Yes | Yes |
| chr20 | 23605963 | G | T | CST9 |  | FIVE_PRIME_FLANK | - | - | - | Yes | Yes | Yes |
| chr20 | 46035472 | C | A | SLC12A5 | c.216C>A | SILENT | - | - | - | Yes | Yes | Yes |
| chr20 | 62289176 | T | G | OSBPL2 | c.e12-31T>G | INTRON | - | - | - | Yes | Yes | Yes |
| chr21 | 36486082 | C | A | CLDN14 | c.e2-24281G>T | INTRON | - | - | - | Yes | Yes | Yes |
| chr21 | 44390976 | G | A | TRPM2 | c.1391G>A | MISSENSE | - | - | - | Yes | Yes | Yes |
| chr22 | 12624318 | G | A | FRG1GP | c.e7-13G>A | RNA | - | - | - | Yes | Yes | Yes |
| chr3 | 5188126 | G | T | EDEM1 | c.321G>T | SILENT | - | - | - | Yes | Yes | Yes |
| chr3 | 27633535 | G | A | ENSG00000271943 | c.e1-185G>A | RNA | - | - | - | Yes | Yes | Yes |
| chr3 | 42864827 | C | A | CYP8B1 | c.e2+8356G>T | INTRON | - | - | - | Yes | Yes | Yes |
| chr3 | 52487634 | C | G | NISCH | c.2142C>G | MISSENSE | - | - | - | Yes | Yes | Yes |
| chr3 | 130593190 | G | GT | COL6A6 | c.e17-9G>GT | INTRON | - | - | - | Yes | Yes | Yes |
| chr4 | 15731686 | G | A | BST1 | c.e9-54G>A | INTRON | - | - | - | Yes | Yes | Yes |
| chr4 | 70197259 | T | C | ODAM | c.e4-15T>C | INTRON | - | - | - | Yes | Yes | Yes |
| chr4 | 89895167 | G | A | MMRN1 | c.196G>A | MISSENSE | - | - | - | Yes | Yes | Yes |
| chr4 | 116599076 | G | A | ACTN4P1 | c.e1-535G>A | RNA | - | - | - | Yes | Yes | Yes |
| chr5 | 41176631 | A | G | C6 | c.1012T>C | MISSENSE | - | - | - | Yes | Yes | Yes |
| chr5 | 45396486 | A | T | HCN1 | c.e4-6T>A | INTRON | - | - | - | Yes | Yes | Yes |
| chr5 | 90658368 | G | T | ADGRV1 | c.e21+90G>T | INTRON | - | - | - | Yes | Yes | Yes |
| chr5 | 90703775 | G | A | ADGRV1 | c.8266G>A | MISSENSE | - | - | - | Yes | Yes | Yes |
| chr5 | 136226307 | C | T | TRPC7 | c.e8+52G>A | INTRON | - | - | - | Yes | Yes | Yes |
| chr5 | 155492343 | A | G | ENSG00000270442 | c.e1+1007A>G | RNA | - | - | - | Yes | Yes | Yes |
| chr5 | 160393893 | T | A | ZBED8 | c.1598A>T | MISSENSE | - | - | - | Yes | Yes | Yes |
| chr5 | 170720331 | G | T | KCNIP1 | c.197G>T | MISSENSE | - | - | - | Yes | Yes | Yes |
| chr6 | 10482938 | G | A | LINC02522 |  | FIVE_PRIME_FLANK | - | - | - | Yes | Yes | Yes |
| chr6 | 18130827 | T | A | TPMT | c.e9+47A>T | INTRON | - | - | - | Yes | Yes | Yes |
| chr6 | 28572052 | G | T | ZBED9 | c.3837C>A | SILENT | - | - | - | Yes | Yes | Yes |
| chr6 | 30697216 | C | T | RPL7P4 | c.e1-319G>A | RNA | - | - | - | Yes | Yes | Yes |
| chr6 | 31548199 | C | T | NFKBIL1 | c.94C>T | MISSENSE | - | - | - | Yes | Yes | Yes |
| chr6 | 31593061 | G | C | NCR3 |  | FIVE_PRIME_FLANK | - | - | - | Yes | Yes | Yes |
| chr6 | 46587215 | C | T | CYP39A1 | c.e10+50G>A | INTRON | - | - | - | Yes | Yes | Yes |
| chr6 | 87215952 | C | T | ZNF292 | c.218C>T | MISSENSE | - | - | - | Yes | Yes | Yes |
| chr6 | 100890235 | G | A | ENSG00000270987 | c.e1-103G>A | RNA | - | - | - | Yes | Yes | Yes |
| chr6 | 104950543 | T | A | LIN28B | c.e2+34T>A | INTRON | - | - | - | Yes | Yes | Yes |
| chr6 | 135455920 | C | T | AHI1 | c.1158G>A | SILENT | - | - | - | Yes | Yes | Yes |
| chr6 | 137706897 | G | A | ENSG00000220412 | c.e1-304C>T | RNA | - | - | - | Yes | Yes | Yes |
| chr7 | 17873571 | G | C | SNX13 | c.710C>G | MISSENSE | - | - | - | Yes | Yes | Yes |
| chr7 | 30632338 | C | T | GARS1 | c.1995C>T | SILENT | - | - | - | Yes | Yes | Yes |
| chr7 | 33604792 | A | T | BBS9 | c.e22-73A>T | INTRON | - | - | - | Yes | Yes | Yes |
| chr7 | 34078974 | C | T | BMPER | c.1196C>T | MISSENSE | - | - | - | Yes | Yes | Yes |
| chr7 | 95435097 | G | T | PON2 |  | FIVE_PRIME_UTR | - | - | - | Yes | Yes | Yes |
| chr7 | 100792533 | G | A | ZAN | c.7841G>A | MISSENSE | - | - | - | Yes | Yes | Yes |
| chr7 | 134987931 | A | G | AGBL3 |  | FIVE_PRIME_UTR | - | - | - | Yes | Yes | Yes |
| chr7 | 142602142 | C | T | TRBV17 | c.126C>T | RNA | - | - | - | Yes | Yes | Yes |
| chr7 | 154986190 | A | C | PAXIP1 | c.e5+2858T>G | INTRON | - | - | - | Yes | Yes | Yes |
| chr7 | 158138337 | C | T | PTPRN2 | c.1089G>A | SILENT | - | - | - | Yes | Yes | Yes |
| chr8 | 66076531 | G | T | DNAJC5B | c.e4-129G>T | INTRON | - | - | - | Yes | Yes | Yes |
| chr8 | 67518075 | C | A | CPA6 | c.e3+28G>T | INTRON | - | - | - | Yes | Yes | Yes |
| chr8 | 78738621 | T | A | IL7 | c.243A>T | MISSENSE | - | - | - | Yes | Yes | Yes |
| chr8 | 90625575 | G | T | TMEM64 |  | THREE_PRIME_UTR | - | - | - | Yes | Yes | Yes |
| chr8 | 95048653 | G | C | NDUFAF6 | c.e13+95G>C | INTRON | - | - | - | Yes | Yes | Yes |
| chr8 | 125044722 | C | G | WASHC5 | c.e21+24G>C | INTRON | - | - | - | Yes | Yes | Yes |
| chr8 | 143721851 | G | A | MAPK15 | c.1429G>A | MISSENSE | - | - | - | Yes | Yes | Yes |
| chr8 | 143869294 | G | A | EPPK1 | c.3960C>T | SILENT | - | - | - | Yes | Yes | Yes |
| chr9 | 977493 | G | C | DMRT3 | c.e1+38G>C | INTRON | - | - | - | Yes | Yes | Yes |
| chr9 | 22451420 | C | T | DMRTA1 | c.1024C>T | MISSENSE | - | - | - | Yes | Yes | Yes |
| chr9 | 32634820 | G | A | TAF1L | c.760C>T | NONSENSE | - | - | - | Yes | Yes | Yes |
| chr9 | 81915460 | T | A | SPATA31D5P | c.e4+68T>A | RNA | - | - | - | Yes | Yes | Yes |
| chr9 | 83977834 | A | G | HNRNPK | c.e4+48T>C | INTRON | - | - | - | Yes | Yes | Yes |
| chr9 | 116618557 | A | G | ASTN2 | c.e18+85T>C | INTRON | - | - | - | Yes | Yes | Yes |
| chr9 | 124857956 | G | A | RPL35 | c.334C>T | MISSENSE | - | - | - | Yes | Yes | Yes |
| chr9 | 136260097 | G | A | CCDC187 | c.e20-22C>T | INTRON | - | - | - | Yes | Yes | Yes |
| chrX | 57390508 | T | G | FAAH2 | c.e7+9479T>G | INTRON | - | - | - | Yes | Yes | Yes |
| chr17 | 26603901 | A | C | Unknown |  | IGR | - | - | - | Yes | Yes | - |
| chr19 | 2250315 | TC | T | AMH | c.e2-22TC>T | INTRON | - | - | - | Yes | Yes | - |
| chr19 | 48286912 | A | T | ZNF114 |  | THREE_PRIME_UTR | - | - | - | Yes | Yes | - |
| chr9 | 72208372 | C | T | GDA | c.e4-2315C>T | INTRON | - | - | - | Yes | Yes | - |
| chr13 | 31758481 | T | C | RXFP2 | c.e2+77T>C | INTRON | - | - | - | Yes | - | Yes |
| chr21 | 8452164 | T | A | ENSG00000280441 | c.e5+540T>A | RNA | - | - | - | Yes | - | Yes |
| chr1 | 148124875 | T | C | NBPF11 | c.e6-24A>G | INTRON | - | - | - | Yes | - | - |
| chr11 | 27806768 | A | T | CBX3P1 | c.e1-228A>T | RNA | - | - | - | Yes | - | - |
| chr2 | 130230106 | T | C | NOC2LP1 | c.e1+727T>C | RNA | - | - | - | Yes | - | - |
| chr11 | 22262116 | TTTAAC | T | ANO5 | c.e16-13TTTAAC>T | INTRON | - | - | - | - | Yes | Yes |
| chr2 | 169128607 | T | TA | LRP2 |  | THREE_PRIME_UTR | - | - | - | - | Yes | Yes |
| chr12 | 111322366 | C | T | CUX2 | c.e18-55C>T | INTRON | - | - | - | - | Yes | - |
| chr16 | 46388705 | C | T | Unknown |  | IGR | - | - | - | - | Yes | - |
| chr7 | 142942627 | C | A | KEL | c.e18+98G>T | INTRON | - | - | - | - | Yes | - |
| chr1 | 6455320 | G | T | ESPN | c.e11-1864G>T | INTRON | - | - | - | - | - | Yes |
| chr11 | 3410441 | C | T | FAM86GP | c.e8-8G>A | RNA | - | - | - | - | - | Yes |
| chr19 | 40950519 | T | C | CYP2B7P | c.e9-141T>C | RNA | - | - | - | - | - | Yes |
| chr19 | 40950524 | C | G | CYP2B7P | c.e9-136C>G | RNA | - | - | - | - | - | Yes |
| chr21 | 8880768 | AAG | A | ENSG00000278931 | c.e9-208AAG>A | RNA | - | - | - | - | - | Yes |
| chrX | 45983552 | C | G | KRT18P68 | c.e1+187C>G | RNA | - | - | - | - | - | Yes |

**Additional file 1: Table S3. Extrachromosomal DNA (ecDNA) cargo genes predicted and classified by Gene-level Circular Amplicon Prediction (GCAP) based on whole exome sequencing (WES) data**

| **Sample** | **Band** | **Ensemble gene id** | **SYMBOL** | **Total copy number** | **Copy number of minor allele** | **Ploidy** | **Background copy number** | **Probability** | **Gene class** |
| --- | --- | --- | --- | --- | --- | --- | --- | --- | --- |
| GBM1_P0 | chr12:q14.1 | ENSG00000037897 | METTL1 | 128 | 39 | 1.98369817 | 4.027117435 | 0.996176124 | circular |
|  |  | ENSG00000111012 | CYP27B1 | 128 | 39 | 1.98369817 | 4.026808867 | 0.996176124 | circular |
|  |  | ENSG00000123297 | TSFM | 128 | 39 | 1.98369817 | 4.023567288 | 0.996176124 | circular |
|  |  | ENSG00000123427 | EEF1AKMT3 | 128 | 39 | 1.98369817 | 4.02750084 | 0.996176124 | circular |
|  |  | ENSG00000135446 | CDK4 | 128 | 39 | 1.98369817 | 4.027365658 | 0.996176124 | circular |
|  |  | ENSG00000135452 | TSPAN31 | 128 | 39 | 1.98369817 | 4.026325478 | 0.996176124 | circular |
|  |  | ENSG00000139266 | MARCHF9 | 128 | 39 | 1.98369817 | 4.026882502 | 0.996176124 | circular |
|  |  | ENSG00000175215 | CTDSP2 | 86 | 26 | 1.98369817 | 4.023653585 | 0.996176124 | circular |
|  |  | ENSG00000257921 |  | 128 | 39 | 1.98369817 | 4.02449203 | 0.996176124 | circular |
|  | chr12:q15 | ENSG00000135679 | MDM2 | 77 | 12 | 1.98369817 | 4.103307008 | 0.996176124 | circular |
|  |  | ENSG00000175782 | SLC35E3 | 77 | 12 | 1.98369817 | 4.090842699 | 0.996176124 | circular |
|  | chr4:q12 | ENSG00000128039 | SRD5A3 | 88 | 10 | 1.98369817 | 3.613055007 | 0.990071118 | circular |
|  |  | ENSG00000134851 | TMEM165 | 88 | 10 | 1.98369817 | 3.610961938 | 0.990071118 | circular |
|  |  | ENSG00000134852 | CLOCK | 88 | 10 | 1.98369817 | 3.60682947 | 0.990071118 | circular |
|  |  | ENSG00000174780 | SRP72 | 69 | 16 | 1.98369817 | 3.610021104 | 0.990071118 | circular |
|  |  | ENSG00000196503 | ARL9 | 69 | 16 | 1.98369817 | 3.610230783 | 0.990071118 | circular |
|  |  | ENSG00000288695 |  | 88 | 10 | 1.98369817 | 3.61286052 | 0.990071118 | circular |
| GBM1_S2_R | chr12:q14.1 | ENSG00000037897 | METTL1 | 89 | 7 | 2.033981685 | 4.027117435 | 0.998362362 | circular |
|  |  | ENSG00000111012 | CYP27B1 | 89 | 7 | 2.033981685 | 4.026808867 | 0.998362362 | circular |
|  |  | ENSG00000123297 | TSFM | 89 | 7 | 2.033981685 | 4.023567288 | 0.998362362 | circular |
|  |  | ENSG00000123427 | EEF1AKMT3 | 89 | 7 | 2.033981685 | 4.02750084 | 0.998362362 | circular |
|  |  | ENSG00000135446 | CDK4 | 89 | 7 | 2.033981685 | 4.027365658 | 0.998362362 | circular |
|  |  | ENSG00000135452 | TSPAN31 | 89 | 7 | 2.033981685 | 4.026325478 | 0.998362362 | circular |
|  |  | ENSG00000139266 | MARCHF9 | 89 | 7 | 2.033981685 | 4.026882502 | 0.998362362 | circular |
|  |  | ENSG00000175215 | CTDSP2 | 19 | 2 | 2.033981685 | 4.023653585 | 0.998362362 | circular |
|  |  | ENSG00000257921 |  | 89 | 7 | 2.033981685 | 4.02449203 | 0.998362362 | circular |
|  | chr12:q15 | ENSG00000135679 | MDM2 | 146 | 3 | 2.033981685 | 4.103307008 | 0.998362362 | circular |
|  | chr4:q12 | ENSG00000128039 | SRD5A3 | 167 | 25 | 2.033981685 | 3.613055007 | 0.993840575 | circular |
|  |  | ENSG00000134851 | TMEM165 | 167 | 25 | 2.033981685 | 3.610961938 | 0.993840575 | circular |
|  |  | ENSG00000134852 | CLOCK | 167 | 25 | 2.033981685 | 3.60682947 | 0.993840575 | circular |
|  |  | ENSG00000174780 | SRP72 | 130 | 36 | 2.033981685 | 3.610021104 | 0.993840575 | circular |
|  |  | ENSG00000196503 | ARL9 | 130 | 36 | 2.033981685 | 3.610230783 | 0.993840575 | circular |
|  |  | ENSG00000288695 |  | 167 | 25 | 2.033981685 | 3.61286052 | 0.993840575 | circular |
| GBM1_B3 | chr12:q14.1 | ENSG00000037897 | METTL1 | 91 | 0 | 2.006255732 | 4.027117435 | 0.998362362 | circular |
|  |  | ENSG00000111012 | CYP27B1 | 91 | 0 | 2.006255732 | 4.026808867 | 0.998362362 | circular |
|  |  | ENSG00000123427 | EEF1AKMT3 | 91 | 0 | 2.006255732 | 4.02750084 | 0.998362362 | circular |
|  |  | ENSG00000135446 | CDK4 | 91 | 0 | 2.006255732 | 4.027365658 | 0.998362362 | circular |
|  |  | ENSG00000135452 | TSPAN31 | 91 | 0 | 2.006255732 | 4.026325478 | 0.998362362 | circular |
|  |  | ENSG00000139266 | MARCHF9 | 91 | 0 | 2.006255732 | 4.026882502 | 0.998362362 | circular |
|  |  | ENSG00000175215 | CTDSP2 | 107 | 6 | 2.006255732 | 4.023653585 | 0.998362362 | circular |
|  | chr12:q15 | ENSG00000135679 | MDM2 | 69 | 3 | 2.006255732 | 4.103307008 | 0.998362362 | circular |
|  |  | ENSG00000175782 | SLC35E3 | 69 | 3 | 2.006255732 | 4.090842699 | 0.998362362 | circular |
|  | chr2:p24.2 | ENSG00000151379 | MSGN1 | 30 | 7 | 2.006255732 | 4.017340665 | 0.995267391 | circular |
|  |  | ENSG00000163029 | SMC6 | 30 | 7 | 2.006255732 | 4.010933404 | 0.995267391 | circular |
|  |  | ENSG00000163032 | VSNL1 | 30 | 7 | 2.006255732 | 4.016022777 | 0.995267391 | circular |
|  |  | ENSG00000178295 | GEN1 | 30 | 7 | 2.006255732 | 4.015349789 | 0.995267391 | circular |
|  |  | ENSG00000197872 | CYRIA | 31 | 7 | 2.006255732 | 4.018340656 | 0.995267391 | circular |
|  | chr4:q12 | ENSG00000128039 | SRD5A3 | 136 | 18 | 2.006255732 | 3.613055007 | 0.993840575 | circular |
|  |  | ENSG00000134851 | TMEM165 | 136 | 18 | 2.006255732 | 3.610961938 | 0.993840575 | circular |
|  |  | ENSG00000134852 | CLOCK | 136 | 18 | 2.006255732 | 3.60682947 | 0.993840575 | circular |
|  |  | ENSG00000174780 | SRP72 | 106 | 32 | 2.006255732 | 3.610021104 | 0.993840575 | circular |
|  |  | ENSG00000196503 | ARL9 | 106 | 32 | 2.006255732 | 3.610230783 | 0.993840575 | circular |
|  |  | ENSG00000288695 |  | 136 | 18 | 2.006255732 | 3.61286052 | 0.993840575 | circular |
| GBM3_P0 | chr17:q11.2 | ENSG00000010244 | ZNF207 | 8 | 2 | 1.886696558 | 3.885992519 | 0.883607745 | circular |
|  |  | ENSG00000108666 | C17orf75 | 8 | 2 | 1.886696558 | 3.881814726 | 0.883607745 | circular |
|  |  | ENSG00000108671 | PSMD11 | 8 | 2 | 1.886696558 | 3.87761714 | 0.883607745 | circular |
|  |  | ENSG00000176749 | CDK5R1 | 8 | 2 | 1.886696558 | 3.881774645 | 0.883607745 | circular |
|  | chr17:q21.32 | ENSG00000002919 | SNX11 | 39 | 7 | 1.886696558 | 4.115195148 | 0.994985819 | circular |
|  |  | ENSG00000004897 | CDC27 | 9 | 4 | 1.886696558 | 4.083370079 | 0.958548307 | circular |
|  |  | ENSG00000005243 | COPZ2 | 39 | 7 | 1.886696558 | 4.108155501 | 0.994985819 | circular |
|  |  | ENSG00000006025 | OSBPL7 | 39 | 7 | 1.886696558 | 4.099592908 | 0.994985819 | circular |
|  |  | ENSG00000082641 | NFE2L1 | 39 | 7 | 1.886696558 | 4.114619406 | 0.994985819 | circular |
|  |  | ENSG00000108433 | GOSR2 | 9 | 4 | 1.886696558 | 4.079566625 | 0.958548307 | circular |
|  |  | ENSG00000108439 | PNPO | 39 | 7 | 1.886696558 | 4.101877563 | 0.994985819 | circular |
|  |  | ENSG00000108465 | CDK5RAP3 | 39 | 7 | 1.886696558 | 4.104247764 | 0.994985819 | circular |
|  |  | ENSG00000108468 | CBX1 | 39 | 7 | 1.886696558 | 4.108374758 | 0.994985819 | circular |
|  |  | ENSG00000141294 | LRRC46 | 39 | 7 | 1.886696558 | 4.10055785 | 0.994985819 | circular |
|  |  | ENSG00000141295 | SCRN2 | 39 | 7 | 1.886696558 | 4.100666468 | 0.994985819 | circular |
|  |  | ENSG00000159111 | MRPL10 | 39 | 7 | 1.886696558 | 4.099641366 | 0.994985819 | circular |
|  |  | ENSG00000167182 | SP2 | 39 | 7 | 1.886696558 | 4.103642056 | 0.994985819 | circular |
|  |  | ENSG00000167183 | PRR15L | 39 | 7 | 1.886696558 | 4.105408837 | 0.994985819 | circular |
|  |  | ENSG00000179673 | RPRML | 9 | 4 | 1.886696558 | 4.087536864 | 0.958548307 | circular |
|  |  | ENSG00000189120 | SP6 | 39 | 7 | 1.886696558 | 4.100446382 | 0.994985819 | circular |
|  |  | ENSG00000262633 |  | 9 | 4 | 1.886696558 | 4.075520034 | 0.958548307 | circular |
|  | chr17:q23.2 | ENSG00000062725 | APPBP2 | 46 | 12 | 1.886696558 | 4.270769117 | 0.994985819 | circular |
|  | chr19:q12 | ENSG00000105171 | POP4 | 12 | 2 | 1.886696558 | 4.296017758 | 0.993094981 | circular |
|  |  | ENSG00000131943 | C19orf12 | 12 | 2 | 1.886696558 | 4.28993337 | 0.993094981 | circular |
|  |  | ENSG00000166289 | PLEKHF1 | 12 | 2 | 1.886696558 | 4.295942379 | 0.993094981 | circular |
|  |  | ENSG00000187135 | VSTM2B | 12 | 2 | 1.886696558 | 4.300590872 | 0.993094981 | circular |
|  | chr4:q12 | ENSG00000072201 | LNX1 | 42 | 2 | 1.886696558 | 3.597518594 | 0.990071118 | circular |
|  |  | ENSG00000109189 | USP46 | 42 | 2 | 1.886696558 | 3.603022024 | 0.990071118 | circular |
|  |  | ENSG00000109220 | CHIC2 | 42 | 2 | 1.886696558 | 3.605947236 | 0.990071118 | circular |
|  |  | ENSG00000128045 | RASL11B | 42 | 2 | 1.886696558 | 3.606942995 | 0.990071118 | circular |
|  |  | ENSG00000128052 | KDR | 42 | 2 | 1.886696558 | 3.61263749 | 0.990071118 | circular |
|  |  | ENSG00000134853 | PDGFRA | 42 | 2 | 1.886696558 | 3.606859461 | 0.990071118 | circular |
|  |  | ENSG00000145216 | FIP1L1 | 42 | 2 | 1.886696558 | 3.603929582 | 0.990071118 | circular |
|  |  | ENSG00000157404 | KIT | 42 | 2 | 1.886696558 | 3.611187722 | 0.990071118 | circular |
|  |  | ENSG00000180613 | GSX2 | 42 | 2 | 1.886696558 | 3.607832969 | 0.990071118 | circular |
|  |  | ENSG00000184178 | SCFD2 | 42 | 2 | 1.886696558 | 3.583937339 | 0.990071118 | circular |
|  |  | ENSG00000226887 | ERVMER34-1 | 42 | 2 | 1.886696558 | 3.608009495 | 0.990071118 | circular |
|  |  | ENSG00000282278 |  | 42 | 2 | 1.886696558 | 3.578955582 | 0.990071118 | circular |
| GBM3_P1 | chr4:q12 | ENSG00000128039 | SRD5A3 | 33 | 7 | 3.76374083 | 3.76374083 | 0.969918728 | circular |
|  |  | ENSG00000163069 | SGCB | 73 | 17 | 3.76374083 | 3.76374083 | 0.969918728 | circular |
|  |  | ENSG00000288695 |  | 33 | 7 | 3.76374083 | 3.76374083 | 0.969918728 | circular |
|  | chr8:q24.13 | ENSG00000173334 | TRIB1 | 81 | 2 | 3.76374083 | 5.436162798 | 0.986187577 | circular |
|  | chr8:q24.21 | ENSG00000136997 | MYC | 81 | 2 | 3.76374083 | 5.816738415 | 0.986187577 | circular |
|  |  | ENSG00000147697 | GSDMC | 81 | 2 | 3.76374083 | 5.434662839 | 0.986187577 | circular |
|  |  | ENSG00000153310 | CYRIB | 81 | 2 | 3.76374083 | 5.407115397 | 0.986187577 | circular |
|  |  | ENSG00000168672 | LRATD2 | 81 | 2 | 3.76374083 | 5.443267857 | 0.986187577 | circular |
|  |  | ENSG00000212993 | POU5F1B | 81 | 2 | 3.76374083 | 5.446913476 | 0.986187577 | circular |
| GBM3_P2 | chr4:q12 | ENSG00000128039 | SRD5A3 | 42 | 6 | 5.307854924 | 5.307854924 | 0.868347526 | circular |
|  |  | ENSG00000163069 | SGCB | 138 | 11 | 5.307854924 | 5.307854924 | 0.868347526 | circular |
|  |  | ENSG00000288695 |  | 42 | 6 | 5.307854924 | 5.307854924 | 0.868347526 | circular |
|  | chr8:q24.13 | ENSG00000173334 | TRIB1 | 149 | 3 | 5.307854924 | 5.436162798 | 0.905112028 | circular |
|  | chr8:q24.21 | ENSG00000136997 | MYC | 149 | 3 | 5.307854924 | 5.816738415 | 0.905112028 | circular |
|  |  | ENSG00000147697 | GSDMC | 149 | 3 | 5.307854924 | 5.434662839 | 0.905112028 | circular |
|  |  | ENSG00000153310 | CYRIB | 149 | 3 | 5.307854924 | 5.407115397 | 0.905112028 | circular |
|  |  | ENSG00000168672 | LRATD2 | 149 | 3 | 5.307854924 | 5.443267857 | 0.905112028 | circular |
|  |  | ENSG00000212993 | POU5F1B | 149 | 3 | 5.307854924 | 5.446913476 | 0.905112028 | circular |

**Additional file 1: Table S4. Amplicon classification profiles predicted by AmpliconSuite-pipeline based on whole genome sequencing (WGS) data**

| **Sample name** | **Amplicon number** | **Amplicon**  **decomposition class** | **ecDNA+**  **status** | **BFB+**  **status** | **Predicted number of distinct**  **(non-overlapping) ecDNA**  **in a single amplicon** |
| --- | --- | --- | --- | --- | --- |
| B3 GBM1 | amplicon1 | Cyclic | Positive | None detected | 2 |
| B3 GBM1 | amplicon2 | Cyclic | Positive | None detected | 1 |
| P2 GBM3 | amplicon1 | Cyclic | Positive | None detected | 1 |
| P2 GBM3 | amplicon2 | Cyclic | Positive | None detected | 1 |

**Additional file 1: Table S5. Classification of ecDNA focal amplification genome context and basic properties and entropy of features predicted by AmpliconSuite-pipeline based on whole genome sequencing (WGS) data**

| **Sample name** | **Amplicon number** | **Feature** | **classification of the ecDNA focal amplification genome context** | **Captured region size (bp)** | **Median feature copy number** | **Max feature copy number** | **Total feature entropy (amplicon complexity score)** | **Decomp entropy (Amount of entropy or diversity captured in the AmpliconArchitect decompositions overlapping this feature)** | **Amp nseg entropy**  **(Amount of entropy or diversity captured by the number of genomic segments overlapping this feature)** |
| --- | --- | --- | --- | --- | --- | --- | --- | --- | --- |
| B3 GBM1 | amplicon1 | ecDNA_1 | Simple circular complex background | 2444776 | 30.41086986 | 33.9267453 | 4.835648955 | 0.466201103 | 4.369447852 |
|  |  | ecDNA_2 | Heavily rearranged unichromosomal | 1389050 | 41.45418698 | 315.0385394 | 6.517710061 | 1.96383317 | 4.553876892 |
|  | amplicon2 | ecDNA_1 | Heavily rearranged unichromosomal | 777983 | 145.0433405 | 158.7529173 | 3.979978599 | 0.291099145 | 3.688879454 |
| P2 GBM3 | amplicon1 | ecDNA_1 | Simple circular complex background | 60768 | 114.3865477 | 226.3085725 | 1.274527998 | 0.581380817 | 0.693147181 |
|  | amplicon2 | ecDNA_1 | Simple circular simple background | 5371642 | 57.81081483 | 62.92926099 | 4.51401488 | 0.48866319 | 4.025351691 |

**Additional file 1: Table S6.** **List of genes present on amplicons predicted by AmpliconSuite-pipeline based on whole genome sequencing (WGS) data**

| **Sample name** | **Amplicon number** | **Feature** | **Gene** | **Gene copy number** | **Truncated** | **Is canonical oncogene** |
| --- | --- | --- | --- | --- | --- | --- |
| B3_GBM1 | amplicon1 | ecDNA_1 | FAM49A | 31.48106519 | None | FALSE |
| B3_GBM1 | amplicon1 | ecDNA_1 | GACAT3 | 30.97546352 | None | FALSE |
| B3_GBM1 | amplicon1 | ecDNA_1 | GEN1 | 30.71041967 | None | FALSE |
| B3_GBM1 | amplicon1 | ecDNA_1 | KCNS3 | 31.16970772 | None | FALSE |
| B3_GBM1 | amplicon1 | ecDNA_1 | MSGN1 | 30.71041967 | None | FALSE |
| B3_GBM1 | amplicon1 | ecDNA_1 | MYCN | 33.9267453 | None | TRUE |
| B3_GBM1 | amplicon1 | ecDNA_1 | MYCNOS | 33.9267453 | 3p | FALSE |
| B3_GBM1 | amplicon1 | ecDNA_1 | RAD51AP2 | 30.05086546 | None | FALSE |
| B3_GBM1 | amplicon1 | ecDNA_1 | SMC6 | 30.71041967 | None | FALSE |
| B3_GBM1 | amplicon1 | ecDNA_1 | VSNL1 | 30.71041967 | None | FALSE |
| B3_GBM1 | amplicon1 | ecDNA_2 | AGAP2 | 92.16709899 | None | TRUE |
| B3_GBM1 | amplicon1 | ecDNA_2 | AGAP2-AS1 | 47.54645779 | None | FALSE |
| B3_GBM1 | amplicon1 | ecDNA_2 | AVIL | 206.065202 | None | FALSE |
| B3_GBM1 | amplicon1 | ecDNA_2 | CDK4 | 92.16709899 | None | TRUE |
| B3_GBM1 | amplicon1 | ecDNA_2 | CPM | 204.7307636 | 5p | FALSE |
| B3_GBM1 | amplicon1 | ecDNA_2 | CTDSP2 | 226.1858474 | None | FALSE |
| B3_GBM1 | amplicon1 | ecDNA_2 | CYP27B1 | 134.1588382 | None | FALSE |
| B3_GBM1 | amplicon1 | ecDNA_2 | EEF1AKMT3 | 134.1588382 | None | FALSE |
| B3_GBM1 | amplicon1 | ecDNA_2 | MARCHF9 | 134.1588382 | None | FALSE |
| B3_GBM1 | amplicon1 | ecDNA_2 | MDM1 | 42.67318144 | 5p | FALSE |
| B3_GBM1 | amplicon1 | ecDNA_2 | MDM2 | 198.9860035 | None | TRUE |
| B3_GBM1 | amplicon1 | ecDNA_2 | METTL1 | 134.1588382 | None | FALSE |
| B3_GBM1 | amplicon1 | ecDNA_2 | NUP107 | 36.83052265 | 5p | FALSE |
| B3_GBM1 | amplicon1 | ecDNA_2 | OS9 | 47.54645779 | 5p | FALSE |
| B3_GBM1 | amplicon1 | ecDNA_2 | RAP1B | 43.77612209 | 5p_3p | FALSE |
| B3_GBM1 | amplicon1 | ecDNA_2 | SLC16A7 | 82.6539731 | 5p | FALSE |
| B3_GBM1 | amplicon1 | ecDNA_2 | SLC35E3 | 36.80034038 | None | FALSE |
| B3_GBM1 | amplicon1 | ecDNA_2 | TAFA2 | 40.41764175 | 3p | FALSE |
| B3_GBM1 | amplicon1 | ecDNA_2 | TSFM | 206.065202 | None | FALSE |
| B3_GBM1 | amplicon1 | ecDNA_2 | TSPAN31 | 92.16709899 | None | FALSE |
| B3_GBM1 | amplicon2 | ecDNA_1 | ARL9 | 136.0911491 | None | FALSE |
| B3_GBM1 | amplicon2 | ecDNA_1 | CLOCK | 145.0433405 | None | FALSE |
| B3_GBM1 | amplicon2 | ecDNA_1 | LNX1 | 2.15550328 | 5p_3p | FALSE |
| B3_GBM1 | amplicon2 | ecDNA_1 | LNX1-AS1 | 2.15550328 | 5p_3p | FALSE |
| B3_GBM1 | amplicon2 | ecDNA_1 | PAICS | 145.395086 | 5p | FALSE |
| B3_GBM1 | amplicon2 | ecDNA_1 | PDCL2 | 144.2527118 | 5p | FALSE |
| B3_GBM1 | amplicon2 | ecDNA_1 | PDGFRA | 150.7828448 | 5p | TRUE |
| B3_GBM1 | amplicon2 | ecDNA_1 | SCFD2 | 147.1724462 | 5p_3p | FALSE |
| B3_GBM1 | amplicon2 | ecDNA_1 | SRD5A3 | 143.149717 | None | FALSE |
| B3_GBM1 | amplicon2 | ecDNA_1 | SRD5A3-AS1 | 143.149717 | None | FALSE |
| B3_GBM1 | amplicon2 | ecDNA_1 | SRP72 | 145.4975677 | None | FALSE |
| B3_GBM1 | amplicon2 | ecDNA_1 | THEGL | 158.7529173 | 3p | FALSE |
| B3_GBM1 | amplicon2 | ecDNA_1 | TMEM165 | 144.8050633 | None | FALSE |
| P2_GBM3 | amplicon1 | ecDNA_1 | SGCB | 114.3865477 | 3p | FALSE |
| P2_GBM3 | amplicon1 | ecDNA_1 | SPATA18 | 114.3865477 | 3p | FALSE |
| P2_GBM3 | amplicon1 | ecDNA_1 | SRD5A3 | 226.3085725 | 5p | FALSE |
| P2_GBM3 | amplicon1 | ecDNA_1 | SRD5A3-AS1 | 226.3085725 | 3p | FALSE |
| P2_GBM3 | amplicon1 | unknown_1 | PDGFRA | 112.8790686 | 5p | TRUE |
| P2_GBM3 | amplicon1 | unknown_1 | SCFD2 | 112.8019774 | 5p_3p | FALSE |
| P2_GBM3 | amplicon2 | ecDNA_1 | ASAP1 | 62.5417513 | None | FALSE |
| P2_GBM3 | amplicon2 | ecDNA_1 | ASAP1-IT1 | 60.05964264 | None | FALSE |
| P2_GBM3 | amplicon2 | ecDNA_1 | ASAP1-IT2 | 60.6483941 | None | FALSE |
| P2_GBM3 | amplicon2 | ecDNA_1 | CASC11 | 62.53269742 | None | FALSE |
| P2_GBM3 | amplicon2 | ecDNA_1 | CASC19 | 53.68746379 | None | FALSE |
| P2_GBM3 | amplicon2 | ecDNA_1 | CASC21 | 58.83286708 | None | FALSE |
| P2_GBM3 | amplicon2 | ecDNA_1 | CASC8 | 58.83286708 | None | FALSE |
| P2_GBM3 | amplicon2 | ecDNA_1 | CCAT1 | 53.68746379 | None | FALSE |
| P2_GBM3 | amplicon2 | ecDNA_1 | CCAT2 | 58.83286708 | None | FALSE |
| P2_GBM3 | amplicon2 | ecDNA_1 | CCDC26 | 59.17714521 | None | FALSE |
| P2_GBM3 | amplicon2 | ecDNA_1 | FAM49B | 62.92926099 | None | FALSE |
| P2_GBM3 | amplicon2 | ecDNA_1 | GSDMC | 62.92926099 | None | FALSE |
| P2_GBM3 | amplicon2 | ecDNA_1 | LRATD2 | 53.6700917 | None | FALSE |
| P2_GBM3 | amplicon2 | ecDNA_1 | MYC | 62.53269742 | None | TRUE |
| P2_GBM3 | amplicon2 | ecDNA_1 | PCAT1 | 53.46308241 | None | FALSE |
| P2_GBM3 | amplicon2 | ecDNA_1 | PCAT2 | 53.68746379 | None | FALSE |
| P2_GBM3 | amplicon2 | ecDNA_1 | POU5F1B | 58.83286708 | None | FALSE |
| P2_GBM3 | amplicon2 | ecDNA_1 | PRNCR1 | 53.68746379 | None | FALSE |
| P2_GBM3 | amplicon2 | ecDNA_1 | PVT1 | 58.1209432 | None | TRUE |
| P2_GBM3 | amplicon2 | ecDNA_1 | TMEM75 | 58.08878323 | None | FALSE |
| P2_GBM3 | amplicon2 | ecDNA_1 | TRIB1 | 59.45744523 | None | TRUE |
